# Virosome-Formulated *Plasmodium falciparum* AMA-1 & CSP Derived Peptides as Malaria Vaccine: Randomized Phase 1b Trial in Semi-Immune Adults & Children

**DOI:** 10.1371/journal.pone.0022273

**Published:** 2011-07-22

**Authors:** Patrick Georges Cech, Thomas Aebi, Mwanajaa Shomari Abdallah, Maxmillian Mpina, Ester Barnabas Machunda, Nicole Westerfeld, Sabine Alexandra Stoffel, Rinaldo Zurbriggen, Gerd Pluschke, Marcel Tanner, Claudia Daubenberger, Blaise Genton, Salim Abdulla

**Affiliations:** 1 Swiss Tropical and Public Health Institute, Basel, Switzerland; 2 University of Basel, Basel, Switzerland; 3 Bagamoyo Research and Training Unit, Ifakara Health Institute, Dar es Salaam, Tanzania; 4 Pevion Biotech AG, Ittigen bei Bern, Switzerland; 5 Infectious Disease Service and Travel Clinic, Lausanne University Hospital, Lausanne, Switzerland; Laboratory of Malaria Immunology and Vaccinology, United States of America

**Keywords:** efficacy, immunogenicity, peptide, malaria, safety, Tanzania, vaccine, virosomes

## Abstract

**Background:**

This trial was conducted to evaluate the safety and immunogenicity of two virosome formulated malaria peptidomimetics derived from *Plasmodium falciparum* AMA-1 and CSP in malaria semi-immune adults and children.

**Methods:**

The design was a prospective randomized, double-blind, controlled, age-deescalating study with two immunizations. 10 adults and 40 children (aged 5–9 years) living in a malaria endemic area were immunized with PEV3B or virosomal influenza vaccine Inflexal®V on day 0 and 90.

**Results:**

No serious or severe adverse events (AEs) related to the vaccines were observed. The only local solicited AE reported was pain at injection site, which affected more children in the Inflexal®V group compared to the PEV3B group (p = 0.014). In the PEV3B group, IgG ELISA endpoint titers specific for the AMA-1 and CSP peptide antigens were significantly higher for most time points compared to the Inflexal®V control group. Across all time points after first immunization the average ratio of endpoint titers to baseline values in PEV3B subjects ranged from 4 to 15 in adults and from 4 to 66 in children. As an exploratory outcome, we found that the incidence rate of clinical malaria episodes in children vaccinees was half the rate of the control children between study days 30 and 365 (0.0035 episodes per day at risk for PEV3B vs. 0.0069 for Inflexal®V; RR  = 0.50 [95%-CI: 0.29–0.88], p = 0.02).

**Conclusion:**

These findings provide a strong basis for the further development of multivalent virosomal malaria peptide vaccines.

**Trial Registration:**

ClinicalTrials.gov NCT00513669

## Introduction

The development of an effective malaria vaccine is regarded as one cornerstone in the fight against this deadly disease and to achieve its eventual elimination [1]. Malaria vaccine development is hindered by the fact that the parasite proceeds through a succession of stages in the human host, with stage specific expression of proteins, a high level of antigen polymorphism, redundancy of essential invasion pathways in host cells, and utilization of a number of immune evasion mechanisms [2]. The lack of an in vitro correlate of protection in malaria and detailed knowledge of the natural host parasite interaction contributes significantly to the slow progress in this field [3]. It is currently assumed that an effective malaria vaccine will likely be comprised of antigens of several developmental stages of the parasite [4].

The circumsporozoite protein (CSP), the major surface protein of the *P. falciparum* sporozoite, has been the focus of numerous efforts to develop a pre-erythrocyte vaccine that aims at prevention of hepatocyte invasion by sporozoites [5]. The CSP forms a dense coat covering the entire surface of the sporozoites, and is critical for sporozoite localization and development of the parasites' liver stage [6]. Antibodies against CSP are primarily directed against the central repeat region [7]. We have developed a synthetic peptide closely resembling the natural conformation of the CSP repeat region [8]. The peptide-phospatidylethanolamine (PE) conjugate is named UK-39 and represents a circularized structure of five NANP repeats [9]. Immunization of rodents with UK-39 coupled to the surface of immuno-potentiating influenza virosomes (IRIV) resulted in high titers of sporozoite cross-reactive antibodies. UK-39 specific IgG inhibited migration and invasion of human hepatocytes by sporozoites providing evidence for protective capacity [10]. The apical membrane antigen 1 (AMA-1), is essential for erythrocyte invasion of *P. falciparum* [11], [12], [13]. It is localized within the apical complex and translocated to the merozoite surface before invasion of erythrocytes commences. It is also expressed in sporozoites [14]. AMA-1-specific antibodies can specifically block the entry of merozoites and sporozoites into erythrocytes and hepatocytes, respectively [14], [15], [16]. A cyclized synthetic peptide of 49 amino acids (named AMA49-C1 as PE conjugate), comprising residues 446–490 of the semi-conserved loop I of domain III has been shown to induce asexual blood stage parasite growth inhibitory antibodies [17].

IRIVs are reconstituted viral coats of influenza viruses lacking the infectious nucleo-capsid RNA but retaining their target cell surface binding and fusogenic activity [18]. They are prepared by detergent removal from a mixture of natural and synthetic phospholipids and influenza surface glycoproteins. The haemagglutinin of the influenza virus is a fusion-inducing membrane glycoprotein, which facilitates antigen delivery to immunocompetent cells. Based on pre-clinical studies, it is generally assumed that during vaccine inoculation, influenza antigen-specific CD4 T-cells provide essential T-cell help for B-cells recognizing synthetic non-influenza peptides coupled to the surface of virosomes [19]. Encapsulated in the virosome lumen antigens may also be used to elicit CD8 T-cell responses. In contrast to live viral vectors like adenovirus, which need the infection of target cells for the induction of immune responses against heterologous antigens, the pre-existing influenza specific immune response did not negatively interfere with the induction of malaria peptide-specific humoral and cellular immune responses [20]. Studies in rodents demonstrated that pre-existing anti-influenza immunity enhances the development of high antibody titers against peptide antigens coupled to IRIVs [19], [21]. There are already two well established commercialized virosomal vaccines: the influenza vaccine Inflexal®V, and the hepatitis A vaccine Epaxal®. These vaccines induce specific immunity without causing non-specific inflammatory response and have therefore an excellent local tolerability in both adults and children [22], [23].

In a Phase 1a clinical trial virosomally formulated UK-39 and AMA49-C1 was well-tolerated in malaria non-immune Caucasian volunteers [24]. Both peptides elicited specific antibody responses in all volunteers immunized through three injections. Combined delivery of both peptides did not interfere with the development of an antibody response to either of the antigens. In a Phase 2a experimental sporozoite challenge trial in malaria non-immune Caucasian volunteers, vaccine-related partial protection against sporozoite challenge was observed [25]. In the same trial, vaccine-induced immune responses were boosted under parasite challenge.

For this Phase 1b trial, UK-39 and AMA49-C1 were coupled to the surface of lyophilizable IRIV formulations [26], rendering the vaccine less sensitive to temperature changes and possible instabilities of components which is particularly important for vaccine application in sub-Saharan Africa [26]. The specific objectives were to demonstrate safety, tolerability and immunogenicity in malaria semi-immune subjects.

## Methods

The protocol for this trial and supporting CONSORT checklist are available as supporting information; see Checklist S1 and Protocol S1.

### Ethics Statement

This study was conducted in compliance with ICH-GCP, the Declaration of Helsinki, and local regulatory requirements. The protocol and all related documents were approved by an independent ethics committee in Switzerland (Ethikkommission beider Basel, EKBB) as well as by the Institutional Review Board of the Ifakara Health Institute and the Medical Research Coordination Committee of the National Institute for Medical Research in Tanzania through the Tanzanian Commission for Science and Technology (COSTECH). The trial was registered at www.ClinicalTrials.gov (NCT00513669).

### Trial design

This was a prospective Phase 1b, single-center, randomized, double-blind, controlled, age-deescalating study. The protocol for this trial and supporting CONSORT checklist are available as supporting information; see and Protocol S1 and Checklist S1.

### Participants

A total of 50 healthy subjects were enrolled; 10 adult male volunteers 18–45 years of age and with a BMI of between 18 and 30, and 40 children of both sexes, 5–9 years of age with a mid-upper arm circumference (MUAC) >12 cm. All subjects, or legal representatives thereof, had given written informed consent. Exclusion criteria were: use of any investigational drug or vaccine within 30 days prior to study start or planned use during the study period; chronic immunosuppressant therapy within 6 months prior to study start; chronic medication; immunosuppressive or immune-deficient condition including HIV infection; history of allergic disease; acute disease at the time of enrolment (defined as the presence of a moderate or severe illness with or without fever); acute or chronic, clinically significant pulmonary, cardiovascular, hepatic or renal functional abnormality; acute or chronic diabetes; history of alcohol consumption and/or intravenous drug abuse.

### Study setting

The study was performed at the Bagamoyo Research and Training Unit of the Ifakara Health Institute (BRTU-IHI) from January 2008 to March 2009. Subjects originated from the area around Bagamoyo town, on the Tanzanian coast, 70 km north of Dar-es-Salaam. Malaria transmission in this area is perennial and almost entirely due to P. falciparum. Insecticide Treated Bednets (ITN) are promoted through a national program. Artemether/lumefantrine (Coartem®, Novartis, Switzerland) is currently the first line treatment for P. falciparum malaria in Tanzania.

### Intervention

**Figure 1:**
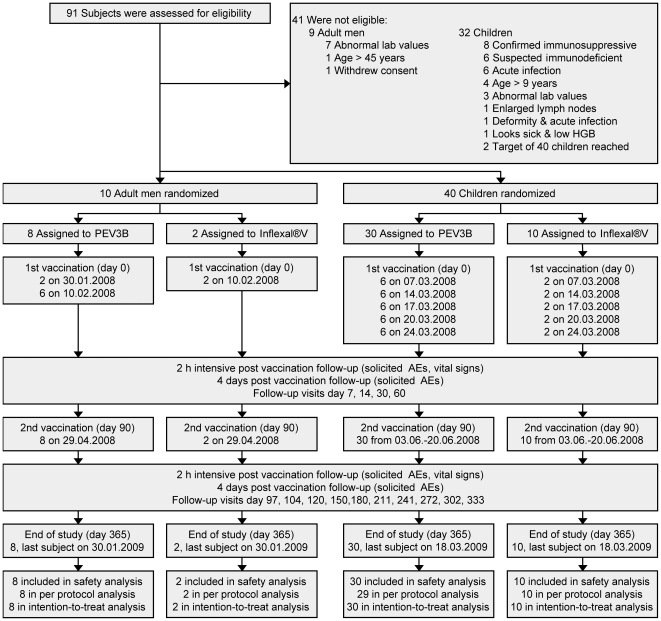
Study flow chart.

The test vaccine PEV3B was composed of 50 µg AMA49-C1 (PEV301T) plus 10 µg UK-39 (PEV302T) peptides formulated in virosomes in phosphate buffered solution pH 7.4 that was subsequently lyophilized. PEV3B lyophilisate was supplied in vials, and reconstituted with 0.6 mL water <4 hours prior to vaccination, of which 0.5 ml were injected. The comparator Inflexal®V is a commercially available virosomal influenza vaccine (Crucell, Switzerland & The Netherlands). The trial vaccines and comparator were administered i.m. in the left arm on day 0 and in the right arm on day 90 (±4 days). With respect to the first vaccination, two adults were immunized before the remaining 8 volunteers with a safety delay of 11 days (Figure 1). After an additional safety observation period of 4 weeks, children were immunized with a safety delay of 1 week between the first two blocks. Subjects showing P. falciparum positive blood smears without any clinical signs or symptoms were cleared from parasites using artemether/lumefantrine prior to immunization. The rationale for this pretreatment was to properly assess AEs following immunization (AEFI) by excluding the potential confounding effect of clinical malaria episodes often developing from asymptomatic parasitaemia [27]. In case subjects presented with acute disease (defined as the presence of a moderate or severe illness with or without fever) or asymptomatic parasitaemia on the planned date of second immunization, administration of the vaccine was delayed upon resolution (end of AE or completion of artemether/lumefantrine treatment).

### Randomization and blinding

Randomization was computer-generated by an independent statistician. The block of 10 adults (PEV3B n = 8, Inflexal®V n = 2) was randomized separately. The 40 children were randomized in blocks of 8 (PEV3B n = 6, Inflexal®V n = 2). A randomization number (1 to 50) was assigned according to the sequence of assignment of the subjects to the study and after inclusion/exclusion criteria were confirmed by the investigator. Vaccines were provided as kits labeled with the randomization number. The trial vaccines and comparator were administered by an independent pharmacist and/or nurse, with the investigators having no access to the vaccination room. Syringes were covered using an opaque foil in order to ensure blinding of the subjects. Investigators and subjects remained blinded until the end of the study and database lock.

### Objectives

The primary objective was evaluation of safety and tolerability of PEV3B in malaria semi-immune subjects. The secondary objective was to measure the magnitude and duration of antibody responses to the malaria specific antigens. Additional exploratory objectives included measuring vaccine-induced cellular immune responses (results to be presented elsewhere) and assessing incidence of clinical episodes of malaria during the follow-up period.

The sample size of this Phase 1b study was determined by the requirement to demonstrate the safety and immunogenicity of the virosome-formulated synthetic peptides. A sample size of 50 volunteers is considered appropriate to estimate incidence rate of frequent AEs and assess immunogenicity with an acceptable accuracy, still allowing for dropouts. Since the target population for malaria vaccines are small children, the adult subgroup included to assess vaccine safety in malaria exposed populations was much smaller than the children group. The adult data are presented for comparison with the children data, but it was not intended to carry out detailed statistical analyses within the adult subgroup. The rationale for an unbalanced allocation of study vaccine and comparator within adult and children subgroups was the main focus on safety and immunogenicity of the experimental malaria vaccine and the established safety profile of the comparator. To rule out that recorded anti-malaria antibody titer increases are caused by cross-reactive immune responses to influenza antigens, the small number of subjects immunized with the comparator influenza vaccine was sufficient.

### Outcomes

#### Safety

Occurrence of solicited local (pain, redness, swelling) and solicited systemic (elevated temperature >37.5°C, headache, fatigue, vertigo) adverse events (AEs) within 4 days after both immunizations was assessed by the physician (see also Figure 1), and general AEs were reported by the subjects throughout the study until Day 365. Intensity of solicited local AEs was graded as follows: pain: 0 = absent, 1 = painful on touch, 2 = painful on movement, 3 = spontaneously painful; redness and swelling: 0 = <5 mm, 1 = 5–20 mm, 2 = 21–50 mm, 3 = > mm. All other AEs were graded and reported according to Common Terminology Criteria for Adverse Events v3.0 (CTCAE). Scheduled haematological and biochemical analyses were performed at screening, baseline (day 0; day of 1^st^ vaccination), on days 7 (±2), 90 (±4; day of 2^nd^ vaccination), and day 7 (±2) after 2^nd^ vaccination.

Safety evaluation included the assessment of the incidence of clinical malaria episodes. A blood sample was taken for parasitological examination by microscopy from all subjects presenting with a history of fever or raised temperature (>37.5°C), irrespective of any other symptoms or sign.

Causality of AEs was categorized as related, probably related, possibly related, unlikely related, and unrelated. All local solicited AEs recorded within 4 days after each vaccination were considered as related to the study vaccine.

#### Immunogenicity

Endpoint titers of anti-AMA49-C1 and anti-UK-39 IgG were measured by ELISA at baseline and on days 30 (±4), 90 (±4; day of 2^nd^ vaccination), 120 (±4), 180 (±7), and day 365 (±14). Procedures for analysis of antibody titers against synthetic peptides using ELISA were described previously [24]. Endpoint titers were determined as last serum dilution where the optical density (OD) of test sera was ≥2× OD of a negative control serum pool of European donors. Swiss TPH and Pevion Biotech AG performed two independent ELISA analyses.

### Statistical methods

#### Safety

The safety analysis included all participants who received at least one vaccination and for whom at least one set of safety follow-up data was available. The proportions of subjects experiencing a given category of AEs were compared using Fisher's exact test for differences between study vaccine and comparator groups.

#### Immunogenicity

The per protocol (PP) population included all participants, who received the two vaccinations in the allowed intervals, attended all the scheduled blood sampling visits in the allowed timeframe, and for whom no major protocol violation was reported. The intention-to-treat (ITT) population included subjects who received at least one vaccination, and for whom at least one blood sample was taken.

Anti-AMA49-C1 and anti-UK-39 IgG endpoint titers were measured by ELISA independently both at Swiss TPH and Pevion Biotech AG. Endpoint titers obtained at Swiss TPH were, on average, slightly higher compared to data from Pevion (p<0.05, sign test, mean/median difference of log [endpoint titers] 0.8/0.7 for AMA49-C1 and 0.3/0 for UK-39), which is an expected variation for repeated ELISA measurements. All statistical analyses were run on the data from each lab separately, as well as on the averaged data set (geometric mean). The identity of the lab, as well as averaging the data, had no influence on the results of the statistical analyses, and the results presented here are for the averaged data set. The exact Wilcoxon test was used to test for differences in endpoint titers between adults and children (at baseline) and between treatment groups (separately for adults and children for each sampling time point). Indices of antibody responses were expressed as the ratio of endpoint titers after immunization with reference to the baseline value (day 0). Wilcoxon's exact test was used to test for differences in indices of antibody responses between treatment groups (separately for adults and children for all time points post first vaccination).

In addition to the above analyses, which were specified in a statistical analysis plan prior to final vaccine accountability and unblinding, the following post-hoc exploratory analyses were performed: 1) The area under the log (antibody titer)-curve above the baseline antibody titer (ΔAUC) was calculated as an integrated measure of antibody response for three intervals: days 0–30, 0–120, and 0–365. ΔAUC values for anti-AMA49-C1 and anti-UK-39 were compared between those subjects who were never tested positive for P. falciparum during the respective interval and those subjects who were tested positive at least once, and Wilcoxon's exact test was used to test for differences. 2) ELISA responders were defined as subjects who seroconverted (from an endpoint titer <50 to ≥50) or showed an at least 4-fold increase in endpoint titer versus baseline. Fisher's exact test was used to test for differences in the proportions of responders between treatment groups (separately for adults and children for all time points post first vaccination). 3) Spearman's rank correlations between anti-AMA49-C1 and anti-UK-39 endpoint titers were computed for each time point in each vaccine group.

#### Malaria morbidity

Time to first or only clinical malaria episodes (confirmed parasitaemia plus clinical symptoms) in children were compared using Kaplan-Meyer failure curves from 30 days post first vaccination until the end of the study (day 365). Difference between failure functions was assessed using the log-rank test, and the hazard ratio was assessed using Cox regression. This analysis had been specified in a statistical analysis plan prior to unblinding. Following the new recommendation of the WHO Malaria Vaccine Advisory Committee [28], we carried out the same analysis separately for two sub-intervals: from day 30 after first vaccination until second vaccination (day 90), and from day 30 after second vaccination (day 120) until the end of the study (day 365). In addition we assessed the treatment effect for multiple episodes of clinical malaria post hoc, using Poisson regression, including time at risk as an offset variable (differences were evaluated using the log likelihood test). Children were not considered susceptible for 28 days after the previous episode, and not considered at risk for a period of 20 days after receiving artemether/lumefantrine treatment and for a period of 7 days after quinine alone [29].

## Results

### Participants

The participant flow is summarized in Figure 1. All 50 subjects completed the study which lasted from 30 January 2008 (first subject immunized) to 18 March 2009 (last subject completed the study). Among adult volunteers (all male) mean age was 32 years in the PEV3B group and 22 years in the Inflexal®V group. In the PEV3B children group 70% of the subjects were male, whereas the gender ratio of the Inflexal®V group was balanced (50%). Mean age, weight, and MUAC were comparable between the children groups (8 years, 19.8 kg, 166 mm for PEV3B, 8 years, 18.8 kg, 162 mm for Inflexal®V). At screening hematology and biochemistry results were generally comparable between the vaccine groups (data not shown). 12 (40%) children in the PEV3B group and 2 (20%) children in the Inflexal®V group had positive blood smears for P. falciparum parasites at screening (asymptomatic carriers). All were cleared from malaria with Coartem® treatment before immunization.

### Safety

All subjects were included in the safety analysis. Solicited (local and general) and unsolicited (general) AEs occurring within 30 days after first and second vaccination are summarized in Table 1. As there were only few AEs reported in adults and the statistical power of test within adult groups was very low due to the small number of subjects, we do not report any results of between-treatment differences in proportion of adult subjects affected by certain categories of AEs.

**Table 1:** Summary table of solicited (local and general) and unsolicited (general) adverse events occurring within 30 days after vaccination.

	Adults				Children			
	PEV3B		Inflexal®V		PEV3B		Inflexal®V	
	N = 8		N = 2		N = 30		N = 10	
**After 1^st^ vaccination (day 0–30)**								
**Local solicited**	**1**	**13%**	**-**	**-**	**2**	**7%**	**1**	**10%**
Pain	1	13%	-	-	2	7%	1	10%
**General solicited**	**-**	**-**	**-**	**-**	**2**	**7%**	**1**	**10%**
Elevated temperature	-	-	-	-	2	7%	1	10%
**General, unsolicited**	**7**	**63%**	**1**	**50%**	**30**	**67%**	**10**	**60%**
**After 2^nd^ vaccination (day 90–120)**								
**Local solicited**	**-**	**-**	**-**	**-**	**3**	**10%**	**5**	**50%**
Pain	-	-	-	-	3	10%	5	50%
**General solicited**	**-**	**-**	**-**	**-**	**4**	**13%**	**1**	**10%**
Elevated temperature	-	-	-	-	2	7%	-	-
Headache	-	-	-	-	2	7%	1	10%
**General, unsolicited**	**2**	**25%**	**-**	**-**	**25**	**57%**	**11**	**70%**
**After 1^st^ and 2^nd^ vaccination (day 0–30 & day 90–120)**								
**Any AE**	**10**	**88%**	**1**	**50%**	**66**	**77%**	**29**	**100%**
**Grade 3 AEs**	**0**	**0%**	**0**	**0%**	**4**	**13%**	**2**	**20%**
**Related AEs** *	**1**	**13%**	**0**	**0%**	**11**	**30%**	**8**	**70%**

Notes: The term “related” refers to AEs judged to be at least possibly related to the vaccine by the clinician. Local solicited AEs comprised pain, redness and swelling, and were by default considered as related to the vaccine. General solicited AEs comprised elevated temperature (>37.5°C), headache, fatigue and vertigo, and all those reported in the table were considered related to the vaccine. For solicited AEs detail on the different AEs are presented in non-bold text.

*comprise all solicited and unsolicited AEs considered as related to the vaccine.

The data on AEs are shown as number of AEs and the proportion of subjects affected.

The only local solicited AEs (monitored from days 0–3 and 90–93) were pain at the injection site, of which all were Grade 1, required no action, and resolved without sequelae. With data combined across both vaccinations the proportion of children reporting pain at the injection site was lower for PEV3B compared to Inflexal®V (see data in Table 1, p = 0.014, Fisher's exact test).

General solicited AEs (monitored from days 0–3 and 90–93) reported in the study were elevated temperature (>37.5°C) and headache; all of these occurred in children (Table 1). Elevated temperature was reported in two (7%) PEV3B children and one (10%) Inflexal®V child after first vaccination, and in two (7%) PEV3B children after second vaccination. Headache was reported in two (7%) PEV3B children and one (10%) Inflexal®V child after second vaccination. Elevated temperature AEs were assessed as “related” (1 event in PEV3B group) or “probably related” (4 events) to the test vaccine, and all headache AEs as “possibly related”. Each of these events was Grade 1 or 2, required no action or treatment with medication, and resolved without sequelae.

All unsolicited AEs (monitored from days 0–365) in this study were considered unrelated to the vaccine and resolved without sequelae.

No statistically significant differences were observed between the children vaccine groups for the proportions of subjects with any AE within 30 days after vaccination, with any Grade 3 AE at any time during the study, or with any related AE at any time during the study (Table 1, p always >0.05, Fisher's exact test).

Eight children (5 PEV3B and 3 Inflexal®V) had serious AEs (SAEs), which were primarily uncomplicated or complicated malaria (definition of complicated malaria was parasitaemia >5000/200 white blood cells). Each of the SAEs required hospitalization and medication, all were considered unlikely related or unrelated to study vaccine, and resolved without sequelae.

No differences in hematology or biochemistry laboratory values between the vaccine groups (adults and children) were observed over the course of the study (data not shown).

### Immunogenicity

The intention-to-treat population comprised 1 subject more than the per protocol population (1 PEV3B child was excluded due to a tetanus toxoid vaccine administered two weeks after second vaccination). Since the exclusion of this subject had no effect on the results of any statistical test, results presented in the following sections are for the intention-to-treat analysis.

**Figure 2:**
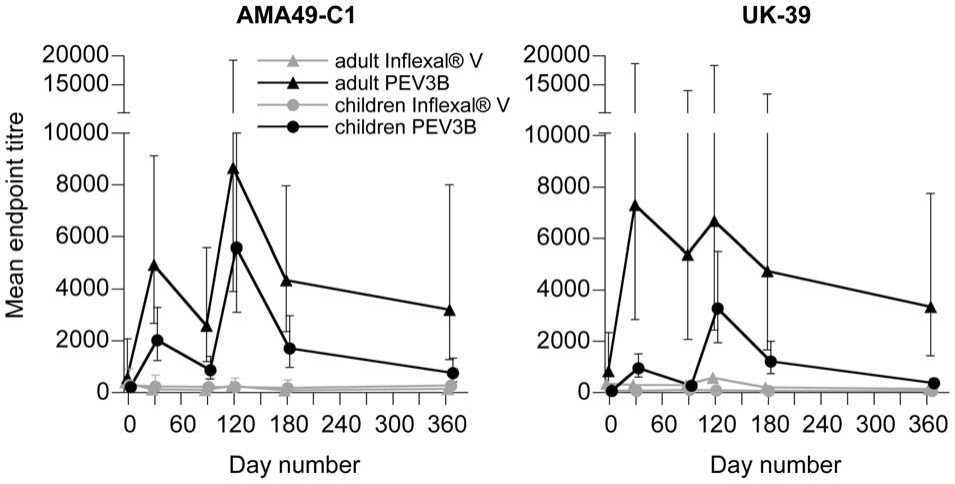
Development of anti-AMA49-C1 and anti-UK-39 IgG ELISA endpoint titres in adults and children immunized with PEV3B or Inflexal®V. Bars indicate 95% confidence intervals of the geometric mean (no bars are shown for adult Inflexal®V, n = 2). Participants were immunized on days 0 and 90 (see arrows).

Within the adult and children subgroups there were no a priori differences in pre-vaccination anti-AMA49 and anti-UK-39 IgG endpoint titers between the vaccine groups (Table 2, see also Figure 2). The pre-inoculation anti-AMA49-C1 IgG endpoint titers were higher in the adults (geometric mean 528; 95%-CI: 168–1657, n = 10) compared to the children (geometric mean 220; 95%-CI: 143–338, n = 40), but this difference was not significant (p = 0.14, exact Wilcoxon test). For pre-vaccination anti-UK-39 IgG, the geometric mean of endpoint titers was significantly higher in adults compared to children, 696 (95%-CI: 304–1594, n = 10) and 54 (95%-CI: 39–73, n = 40), respectively (p<0.0001, exact Wilcoxon test).

**Table 2:** Geometric means and corresponding 95% confidence intervals of anti-AMA-49-C1 and anti-UK-39 IgG ELISA endpoint titres, and the rate of responders (in %) among subjects.

	Adults			Children		
	PEV3B	Inflexal®V	p-value	PEV3B	Inflexal®V	p-value
	N = 8	N = 2		N = 30	N = 10	
**AMA49-C1**						
Baseline	566	400	0.694	202	283	0.571
	155–2067	-		127–323	88–910	
Day 30	4935	119	0.036	2016	230	<0.001
	2669–9125	-		1234–3292	78–676	
	75%	0%	0.133	80%	0%	<0.001
Day 90	2577	100	0.035	857	207	0.005
	1189–5584	-		521–1410	80–533	
	63%	0%	0.444	57%	10%	0.013
Day 120	8667	238	0.034	5572	200	<0.001
	3909–19218	-		3106–9994	72–558	
	75%	50%	1.000	87%	0%	<0.001
Day 180	4334	84	0.034	1695	180	<0.001
	2358–7964	-		967–2972	67–483	
	63%	0%	0.444	70%	10%	0.002
Day 365	3200	141	0.036	746	264	0.077
	1280–8000	-		422–1320	96–725	
	75%	50%	1.000	50%	20%	0.145
**UK-39**						
Baseline	835	336	0.426	49	68	0.353
	298–2346	-		35–70	31–153	
Day 30	7288	283	0.035	951	59	<0.001
	2843–18681	-		602–1502	26–136	
	63%	0%	0.444	93%	10%	<0.001
Day 90	5382	283	0.035	267	87	0.028
	2072–13981	-		178–400	32–238	
	63%	0%	0.444	73%	10%	0.001
Day 120	6683	566	0.067	3275	81	<0.001
	2437–18330	-		1947–5507	31–212	
	63%	0%	0.444	97%	10%	<0.001
Day 180	4726	200	0.036	1213	64	<0.001
	1664–13424	-		736–1997	32–127	
	50%	0%	0.467	87%	10%	<0.001
Day 365	3342	141	0.036	356	54	<0.001
	1442–7743	-		229–554	23–123	
	50%	0%	0.467	83%	10%	<0.001

Notes: For Inflexal®V adults no confidence interval for mean endpoint titers are shown because N = 2. ELISA responders were defined as subjects who seroconverted (from an endpoint titer <50 to ≥50) or showed an at least 4-fold increase in endpoint titer versus baseline. P-values are given for tests for differences between PEV3B and Inflexal®V within adult and children subgroups at the respective time point (exact Wilcoxon test for endpoint titers, Fisher's exact test for the proportion of responders).

The antibody endpoint titers for AMA49-C1 and UK-39 in the PEV3B groups increased in response to the vaccinations as measured 30 days after the first (day 30) and second (day 120) immunization, whereas in the Inflexal®V groups the endpoint titers for both antigens remained at background levels (Figure 2). However, on days 90 and 180 these titers had declined in the PEV3B vaccinees (Figure 2). Endpoint titers were significantly higher in the PEV3B vaccinees than in the Inflexal®V control group at all time points, except at day 120 for UK-39 in adults and day 365 for AMA49-C1 in children (Table 2). In the PEV3B group, endpoint titers were on average higher in adults than in children, and this difference was significant at most sampling time points (compare CIs in Table 2).

**Figure 3:**
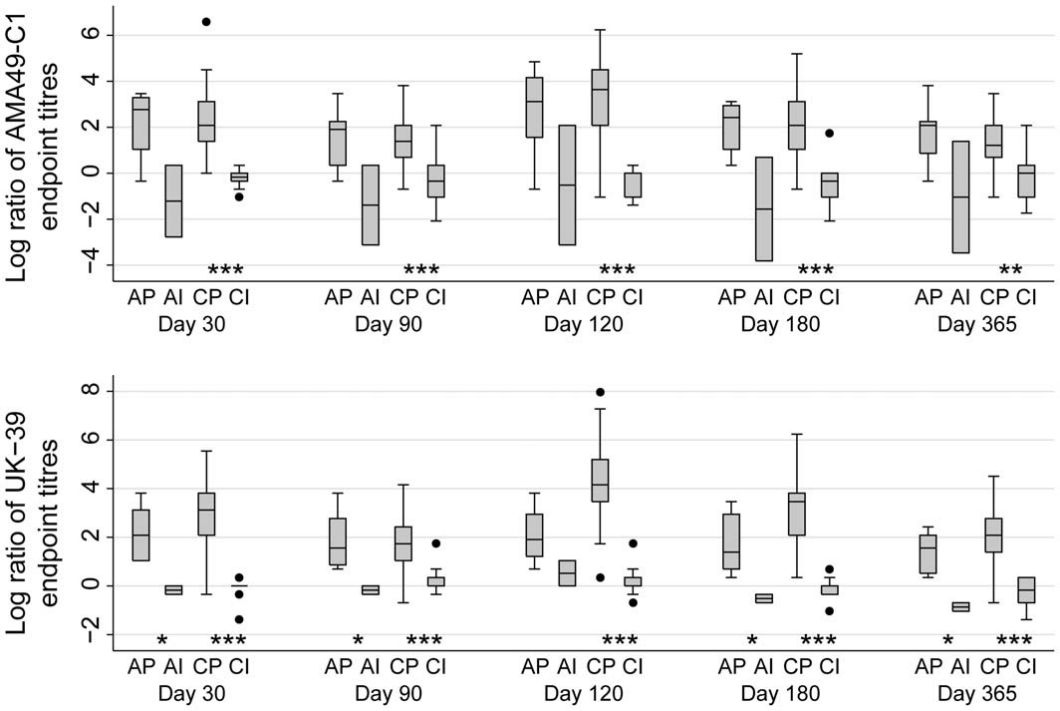
Box-plots of the logarithms of the index of response (ratios of anti-AMA49-C1 and anti-UK-39 IgG ELISA endpoint to baseline titers) for samples taken at day 30, 90, 120, 180, and 365. **AP: adults PEV3B, AI: adults Inflexal®V, CP: children PEV3B, CI: children Inflexal®V.** At all time points the logarithm of the index of response of AP and CP groups were significantly >0 (index of response >1, p<0.05). This was never observed for the AI and CI groups. Symbols indicate the level of significance for differences in the index of response between PEV3B and Inflexal®V groups within adults and children subjects (exact Wilcoxon test, *: p<0.05, **: p<0.01, ***: p<0.001).

In PEV3B vaccinees, the average index of response for AMA49-C1 (ratio of endpoint to baseline titers) ranged from 4.6 (day 90) to 15.3 (day 120) in adults and from 3.7 (day 365) to 27.5 (day 120) in children. For UK-39, the index of response ranged from 4.0 (day 365) to 8.7 (day 30) among adults and from 5.4 (day 90) to 66.3 (day 120) among children (Figure 3). In both adults and children PEV3B vaccinees, the average index of response was >1 for both antigens at all sampling time points (Figure 3). The proportion of responders to either antigen was significantly higher in PEV3B than in Inflexal®V children, except for AMA49-C1 at day 365 (Table 2).

Anti-AMA49-C1 and anti-UK-39 IgG endpoint titers were significantly correlated with each other in PEV3B children at all sampling points including baseline (Spearman's rho ρ = 0.50–0.61; p always <0.01). For the 8 PEV3B adults, the correlation was not significant at any of the sampling points (ρ = −0.51–0.56; p always >0.15). In PEV3B vaccinated children, Spearman's rho was maximal at day 30 and day 120, indicating that the kinetics of antibody responses to AMA49-C1 and UK-39 in response to vaccination correlated with each other (data not shown).

### Malaria morbidity

**Figure 4:**
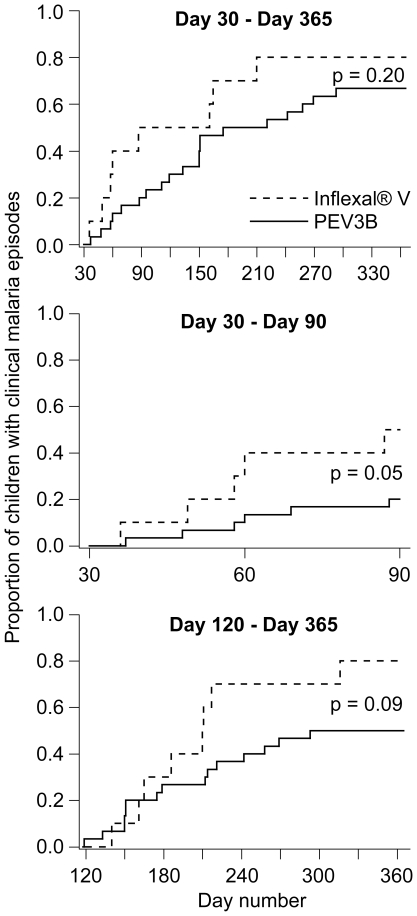
Attack rates of first or only clinical malaria episodes in vaccinated children. Kaplan-Meyer failure functions are shown for the intervals from 30 days after first vaccination (day 30) until the end of the study (day 365), from 30 days after first vaccination until second vaccination (day 90), and from 30 days after second vaccination (day 120) until study end (day 365). P-values for differences in failure functions between treatment groups are given (log-rank test). Note: the failure function in the third panel is not identical with the corresponding part of the failure function in the first panel, because of the occurrence of multiple events in the same individual during the course of the study.

The proportion of children who experienced one or several episode(s) of malaria from the first peak of endpoint titers (day 30) until the end of the follow-up period (day 365) tended to be lower in the PEV3B group than in the Inflexal®V group (67% versus 80% respectively). However, the estimated failure functions were not significantly different (incidence rate of first or only clinical malaria episode per child-day IR^first ep^  = 0.00514 for Inflexal®V and 0.00306 for PEV3B, p = 0.20; hazard ratio HR^first ep^ and 95%-CI  = 0.58 [0.24–1.36]) (Figure 4). For the period between 30 days after first vaccination until the second vaccination, the proportion of children with at least one malaria episode was lower in the PEV3B group than in the Inflexal®V group (28% versus 73%). The difference in the corresponding failure functions was at the threshold of statistical significance (IR^first ep^  = 0.00676 for Inflexal®V and 0.00238 for PEV3B, p = 0.05; HR^first ep^  = 0.31 [0.09–1.09]) (Figure 4). For the period from the highest measured levels of antibody endpoint titers (30 days post second immunization, day 120) until the end of the study (day 365) the proportion of children with at least one malaria episode was lower in the PEV3B group than in the Inflexal®V group (50% versus 80%). The difference in the failure functions was marginally significant (IR^first ep^  = 0.00342 for Inflexal®V and 0.00179 for PEV3B respectively, p = 0.09; HR^first ep^  = 0.48 [0.20–1.15]) (Figure 4). The failure function for PEV3B children in the first panel of Figure 4 shows a marked reduction of malaria events between study days 150 and 220 which corresponds to the lower malaria transmission in the dry period from mid-August to end of October.

The number of clinical malaria episodes per child subject from day 30 after first vaccination until the end of the follow-up period (day 365) was significantly lower in PEV3B vaccinees compared to the control group (IR  = 0.0035 per day at risk for PEV3B and 0.0069 for Inflexal®V, p = 0.02). This corresponds to a reduction of 50% (rate ratio RR  = 0.50, 95%-CI  = 0.29–0.88).

For two adult subjects a clinical episode of malaria was reported – both in the PEV3B group. Thus the proportion of adults affected by malaria was lower than in children. This observation is in line with the increased disease resistance of the adult population to malaria, which therefore is not the target group for a malaria vaccine.

### Correlation between immunogenicity and malaria morbidity

**Figure 5:**
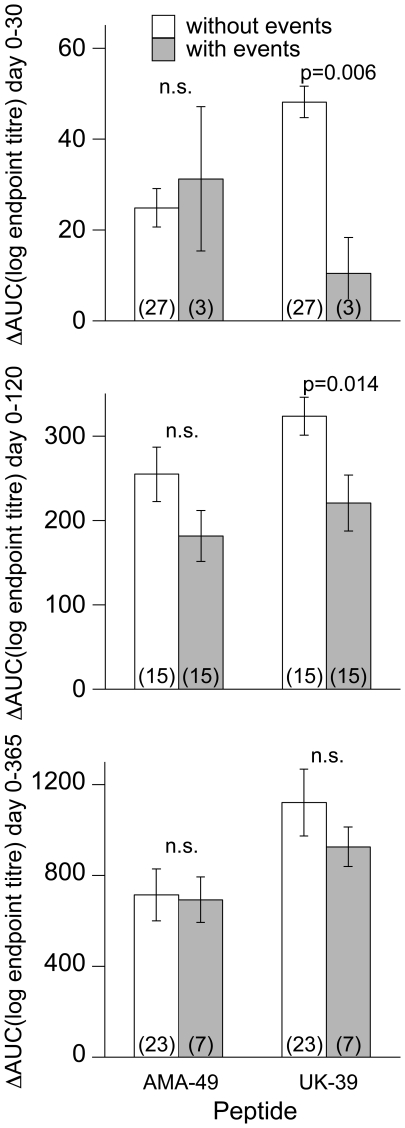
Comparison of the integrated antibody responses against AMA49-C1 and UK-39 with the detection of malaria parasitaemia in PEV3B children during time periods Day 0–30, Day 0–120 and Day 0–365. ΔAUC was calculated as the area under the log (antibody titer)-curve above the baseline antibody titer. ΔAUC values were compared between those PEV3B children who did not have positive smears for *P. falciparum* and those PEV3B children who were tested positive for parasites using Wilcoxon's test. The numbers (in brackets) in the bars indicate the number of PEV3B in each of the groups compared.

Among PEV3B children, those diagnosed with P. falciparum parasitaemia (i.e. asymptomatic cases as well as clinical episodes) during the study periods from day 0–30, day 0–120 and day 0–365 showed a lower integrated antibody response for UK-39 compared to those who remained undiagnosed for P. falciparum parasitaemia (Figure 5). This difference between the two groups of children was statistically significant for the periods day 0–30 and day 0–120 (Figure 5), indicating that UK-39-binding antibodies contribute to prevention of malaria infection. During the study periods from day 0–120 and day 0–365, integrated antibody response for AMA49-C1 was also higher in children remaining undiagnosed for parasitaemia. However, for AMA49-C1 these differences were not statistically significant.

## Discussion

Phase 1a and 2a clinical trials conducted with virosomal formulations of UK-39 and AMA49-C1 in malaria non-immune Caucasian volunteers [24], [25] have yielded encouraging results, but did not address safety and immunogenicity of the vaccine formulation in malaria semi-immune subjects.

The two commercialized virosome-based vaccines (Epaxal® and Inflexal®V) have already demonstrated the excellent safety profile of the virosomal antigen delivery system. In the present trial, local reactogenicity was minimal with only mild or moderate pain and no grade 3 AE related to the vaccine reported. All SAEs were considered unlikely or unrelated to the study vaccine and all resolved without sequelae. This excellent reactogenicity profile contrasts with results obtained with recombinant malaria proteins formulated in ASO2A, ASO1E or Montanide ISA 720 & 51, or DNA vaccines in viral vectors, which show much higher rates of grade 2 and 3 AEs [30], [31], [32], [33].

PEV3B elicited IgG responses to both target antigens in semi-immune adults and children, with the highest titers generally observed 30 days after the second immunization. 275 days after the second immunization (day 365) endpoint titers against both target antigens were still higher than baseline titers. In children, the index of response to UK-39 tended to be stronger compared to that to AMA49-C1, which was primarily due to the lower baseline endpoint titers for UK-39. In adults, responses to both antigens were comparable.

In this Phase 1b trial, malaria morbidity was not the primary outcome, thus the design was not powered to assess efficacy. However, the analysis of time to event for first or only episode of clinical malaria in children suggests that the PEV3B had a protective effect. There was no statistically significant difference in incidence rate of first or only clinical malaria episode for the period from 30 days after the first vaccination until the end of the study on day 365. For two separate intervals, from day 30 after first vaccination until second vaccination, and from day 30 after second vaccination until the end of the study, however, the reduction of incidence rate of first or only clinical malaria episode by 70% and 50% respectively was approaching statistical significance. These results, which look inconsistent at first glance, are explained by the significantly higher rate of malaria episodes in control subjects compared to PEV3B vaccinees. Multiple events in the same subject are not captured in a conventional time-to-first-event Kaplan-Meier graph. Data on the total number of episodes are a better measure for the disease burden from a public health perspective compared to first episode data [28]. In this study, incidence rate of clinical malaria episodes in children vaccinated with PEV3B was half the rate of the control group, and this difference was statistically significant. A 50% efficacy to reduce clinical malaria in children is equivalent to the protection conferred by what is considered the most advanced malaria vaccine to date, namely RTS,S, which indicates that the virosomal platform with synthetic peptides represents a very promising technology. For the analysis of data on multiple malaria episodes we used the same methodology as Sacarlal et al. in their RTS,S trial in Mozambican children [29]. However, multiple episodes in the same subject are not independent from each other and the analysis of such data requires more statistical research [28]. The exploratory efficacy results from the present study are in line with the measured antibody responses, and the indications for vaccine-induced impact on asexual parasite blood stage multiplication in the Phase 2a sporozoite challenge trial [25].

In the children PEV3B group, subjects with at least one episode of P. falciparum parasitaemia in the study periods day 0–30 and day 0–120 showed a significantly lower average response in antibody titers against UK-39 for the corresponding period. For anti-AMA49-C1 the differences in response of antibody titers related to incidence of parasitaemia were not statistically significant. This may indicate that the induced antibody response to UK-39 is a key component of the possible protective effect of PEV3B, and raises the question whether there is a link to the pattern of differences in background antibody levels between the highly immune adult population and children. A close correlation between anti-UK-39 IgG titers assessed in ELISA, immunofluorescence and Western blotting analysis using sporozoites and sporozoite lysates, respectively, has already been demonstrated [10]. The anti-UK-39 antibody titers showed a close correlation to in vitro inhibition of sporozoite migration and invasion of hepatocytes [10].

The most advanced subunit malaria vaccine to date, RTS,S, is currently tested in a phase III clinical trial (NCT00866619) and is based on a fusion protein of part of the CSP of P. falciparum clone 3D7 (amino acids 207 – 395) with the hepatitis B surface antigen [34].

Studies with RTS,S in an endemic area of Mozambique showed that vaccination of children aged 1–4 years induced partial protection against infection and clinical malaria including severe disease [35]. RTS, S elicits strong humoral responses to the B cell epitopes located in the central repeat region [34]. Evidence accumulating over the years indicate strongly that high antibody titres against the CSP correlate with protection [36]. These CSP-specific antibodies may contribute to elimination of sporozoites and infected hepatocytes by different mechanisms like neutralization of sporozoites by inhibiting gliding motility and cell traversal, Fc receptor-mediated engulfment of sporozoites, Fc-receptor dependent lysis by NK and NKT cells or by activation of the complement system after antibody binding.

Since IRIVs represent a modular antigen delivery system, they are an ideal platform to evaluate individual antigens separately, which can subsequently be combined with other components to a multi-component subunit vaccine [24]. IRIV-based formulations of additional malaria antigens have been optimized in pre-clinical studies [21], [37], [38]. After individual clinical profiling they could be added to the bivalent formulation tested in the present trial to form a multivalent malaria vaccine. Our results indicate that by conducting well-designed combined Phase 1b/2 trials in malaria endemic regions, safety and immunogenicity can be assessed and efficacy data can be obtained. These trials thus can represent an important element in the rational design of an efficient malaria multi-component vaccine, and could reduce time and cost of development.

## Supporting Information

Checklist S1 **CONSORT checklist.**
(PDF)

Protocol S1 **Trial protocol.**
(PDF)
