# Supplementary material for: Virosome-Formulated Plasmodium falciparum AMA-1 & CSP Derived Peptides as Malaria Vaccine: Randomized Phase 1b Trial in Semi-Immune Adults & Children
Source: PLoS One. 2011 Jul 22;6(7):e22273. doi: 10.1371/journal.pone.0022273 (PMC3142124; doi:10.1371/journal.pone.0022273)
Supplement: Protocol S1 — Trial protocol. (PDF) [file pone.0022273.s002.pdf]

Swiss Tropical Institute
Institut Tropical Suisse
Schweizerisches Tropeninstitut

Confidential

CLINICAL TRIAL

Protocol number. PMAL03 v02
Amended Protocol version: August 15, 2007

A phase Ib double-blind randomized controlled age-deescalating trial of two virosome formulated anti-malaria vaccine components (PEV301T and PEV302T) administered in combination (PEV3B) to healthy semi-immune Tanzanian volunteers

Swiss Tropical Institute, Basel, Switzerland
Ifakara Health research and Development Center, Tanzania
Pevion Biotech Ltd, Bern

Confidentiality Statement

This document contains information, which is the property of the institutions and company involved and therefore is provided to you in confidence for review by you, your staff, an applicable ethics committee / institutional review board and regulatory authorities. It is understood that this information will not be disclosed to others without written approval from the investigators, except to the extent necessary to obtain informed consent from those persons (or their legal guardian) to whom the vaccine may be administered.

PROTOCOL NUMBER:	PMAL03, Amended Protocol (v02)
DATE OF PROTOCOL:	15.8.2007
PROTOCOL TITLE:	A phase Ib double-blind randomized controlled age-deescalating trial of two virosome formulated anti-malaria vaccine components (PEV301T and PEV302T) administered in combination (PEV3B) to healthy semi-immune Tanzanian volunteers
SPONSOR	Pevion Biotech Ltd., Berne

RESPONSIBLE STUDY PERSONNEL:

Co-PRINCIPAL INVESTIGATOR (principal protocol author)
Name: Dr. Blaise Genton MD PhD DTMH
Address: Ifakara Health Research and Development Center (IHRDC)
Po Box 78373, Dar Es Salaam
Swiss Tropical Institute
Phone: +255-786-19 00 69 (Mobile), +255 22 277 47 56
Fax: +255 22 277 17 14
e-mail blaise.genton@unibas.ch

Co-PRINCIPAL INVESTIGATOR
Name: Dr. Salim Abdulla MD PhD
Address: Ifakara Health Research and Development Center (IHRDC)
Phone: +255 22 277 47 56
Fax: +255 22 277 17 14
e-mail salim.abdulla@gmail.com

SPONSOR and IMMUNOLOGY LABORATORY, PEVION BIOTECH LTD.
Name: Dr. Rinaldo Zurbriggen
Address: Pevion Biotech Ltd., Rehhagstrasse 79, CH-3018 Berne
Phone: +41-31-980-6417
Fax: +41-31-980-6618
e-mail rinaldo.zurbriggen@pevion.com

SCIENTIFIC ADVISOR IMMUNOLOGY, IMMUNOLOGY LABORATORY
Department of Medical Parasitology & Infection Biology, Swiss Tropical Institute
Name: Prof. Dr. Gerd Pluschke
Address: Swiss Tropical Institute, Socinstrasse 57, CH 4002 Basel
Phone: +41-61-284 8235
Fax: +41-61-271 8654
e-mail gerd.pluschke@unibas.ch

STATISTICIAN
Ifakara Health Research and Development Center
Name: Ali Mohamed Ali
Address: Ifakara Health Research and Development Center (IHRDC)
Po Box 78 373
Dar Es Salaam, Tanzania (Republic of)
Phone: +255 22 277 47 56; +255 23 2440065
Fax: +255 22 277 47 14; +255 23 2440064

CLINICAL RESEARCH ORGANISATION
Swiss Centre for International Health, Swiss Tropical Institute
Head of PMU at SCIH

Name: Dr. Christian Burri
Address: SCIH, Swiss Tropical Institute, Socinstrasse 57, CH 4002 Basel
Phone: +41-61-225 2661
Fax: +41-61-225 2678
e-mail christian.burri@unibas.ch

STUDY COORDINATOR CRO

Name: Dr. Hermann Garden
Address: SCIH, Swiss Tropical Institute, Socinstrasse 57, CH 4002 Basel
Phone: +41-61-225 2666
Fax: +41-61-225 2678
e-mail hermann.garden@unibas.ch

DATA MANAGEMENT
Ifakara Health Research and Development Center

Name: Honorati Masanja
Address: Po Box 78373
Phone: +255 22 277 17 56
Fax: +255 22 277 17 14
e-mail hmasanja@ihrdc.or.tz

Signature Page

Co-PRINCIPAL INVESTIGATOR

Name:<br>Address:	Blaise Genton, MD, PhD, DTM&H<br>Ifakara Health Research and Development Center (IHRDC)<br>Po Box 78 373<br>Dar Es Salaam, Tanzania (Republic of)
Phone:<br>Fax:	+255 786 19 00 69; +255 22 277 47 56<br>+255 22 277 47 14

I, the undersigned, have reviewed this Protocol, including Appendices and I will conduct the clinical study as described and will adhere to ICH-GCP Guidelines and all the ethical and regulatory considerations stated. I have read and understood the contents of the Investigator Brochure.

Date/Place______________________________Signature__________________________

Co-PRINCIPAL INVESTIGATOR

Name:<br>Address:	Salim Abdulla MD, PhD<br>Ifakara Health Research and Development Center (IHRDC)<br>Po Box 78 373<br>Dar Es Salaam, Tanzania (Republic of)
Phone:<br>Fax:	Mobile +255 754 744 555; +255 22 277 47 56; +255 23 2440065<br>+255 22 277 47 14; +255 23 2440064

I, the undersigned, have reviewed this Protocol, including Appendices and I will conduct the clinical study as described and will adhere to ICH-GCP Guidelines and all the ethical and regulatory considerations stated. I have read and understood the contents of the Investigator Brochure.

Date/Place______________________________ Signature__________________________

SPONSOR

Name:<br>Address:	Rinaldo Zurbriggen, PhD<br>Pevion Biotech Ltd., Rehhagstrasse 79<br>CH-3018 Bern, Switzerland
Phone:<br>Fax:	+41 31 980 64 17<br>+41 31 980 66 18

I, the undersigned, have reviewed the protocol and confirm that the protocol, including Appendices, follows the current ICH-GCP guidelines.

Date/Place______________________________ Signature__________________________

STUDY MANAGEMENT AND MONITORING

Name:<br>Address:	Christian Burri, PD Dr.<br>SCIH, Swiss Tropical Institute, Socinstrasse 57, CH 4002 Basel
Phone:<br>Fax:	+41 61 225 26 61<br>+41 61 225 26 78

I, the undersigned, have reviewed this Protocol, including Appendices and I will conduct the clinical study as described and will adhere to ICH-GCP and all the ethical and regulatory considerations stated.

Date/Place______________________________ Signature__________________________

Signature Page

Co-PRINCIPAL INVESTIGATOR

Name:<br>Address:	Blaise Genton, MD, PhD, DTM&H<br>Ifakara Health Research and Development Center (IHRDC)<br>Po Box 78 373<br>Dar Es Salaam, Tanzania (Republic of)
Phone:<br>Fax:	+255 786 19 00 69; +255 22 277 47 56<br>+255 22 277 47 14

I, the undersigned, have reviewed this Protocol, including Appendices and I will conduct the clinical study as described and will adhere to ICH-GCP Guidelines and all the ethical and regulatory considerations stated. I have read and understood the contents of the Investigator Brochure.

Date/Place Dar Es Salaam 17.08.07 Signature [signature]

Co-PRINCIPAL INVESTIGATOR

Name:<br>Address:	Salim Abdulla MD, PhD<br>Ifakara Health Research and Development Center (IHRDC)<br>Po Box 78 373<br>Dar Es Salaam, Tanzania (Republic of)
Phone:<br>Fax:	Mobile +255 754 744 555; +255 22 277 47 56; +255 23 2440065<br>+255 22 277 47 14; +255 23 2440064

I, the undersigned, have reviewed this Protocol, including Appendices and I will conduct the clinical study as described and will adhere to ICH-GCP Guidelines and all the ethical and regulatory considerations stated. I have read and understood the contents of the Investigator Brochure.

Date/Place 17.08.07 DAR ES-SALAAM Signature [signature]

SPONSOR

Name:<br>Address:	Rinaldo Zurbriggen, PhD<br>Pevion Biotech Ltd., Rehhagstrasse 79<br>CH-3018 Bern, Switzerland
Phone:<br>Fax:	+41 31 980 64 17<br>+41 31 980 66 18

I, the undersigned, have reviewed the protocol and confirm that the protocol, including Appendices, follows the current ICH-GCP guidelines.

Date/Place____________________ Signature____________________

STUDY MANAGEMENT AND MONITORING

Name:<br>Address:	Christian Burri, PD Dr.<br>SCIH, Swiss Tropical Institute, Socinstrasse 57, CH 4002 Basel
Phone:<br>Fax:	+41 61 225 26 61<br>+41 61 225 26 78

I, the undersigned, have reviewed this Protocol, including Appendices and I will conduct the clinical study as described and will adhere to ICH-GCP and all the ethical and regulatory considerations stated.

Date/Place____________________ Signature____________________

Signature Page

Co-PRINCIPAL INVESTIGATOR

Name:<br>Address:	Blaise Genton, MD, PhD, DTM&H<br>Ifakara Health Research and Development Center (IHRDC)<br>Po Box 78 373<br>Dar Es Salaam, Tanzania (Republic of)
Phone:<br>Fax:	+255 786 19 00 69; +255 22 277 47 56<br>+255 22 277 47 14

I, the undersigned, have reviewed this Protocol, including Appendices and I will conduct the clinical study as described and will adhere to ICH-GCP Guidelines and all the ethical and regulatory considerations stated. I have read and understood the contents of the Investigator Brochure.

Date/Place________________ Signature________________

Co-PRINCIPAL INVESTIGATOR

Name:<br>Address:	Salim Abdulla MD, PhD<br>Ifakara Health Research and Development Center (IHRDC)<br>Po Box 78 373<br>Dar Es Salaam, Tanzania (Republic of)
Phone:<br>Fax:	Mobile +255 754 744 555; +255 22 277 47 56; +255 23 2440065<br>+255 22 277 47 14; +255 23 2440064

I, the undersigned, have reviewed this Protocol, including Appendices and I will conduct the clinical study as described and will adhere to ICH-GCP Guidelines and all the ethical and regulatory considerations stated. I have read and understood the contents of the Investigator Brochure.

Date/Place________________ Signature________________

SPONSOR

Name:<br>Address:	Rinaldo Zurbriggen, PhD<br>Pevion Biotech Ltd., Rehhagstrasse 79<br>CH-3018 Bern, Switzerland
Phone:<br>Fax:	+41 31 980 64 17<br>+41 31 980 66 18

I, the undersigned, have reviewed the protocol and confirm that the protocol, including Appendices, follows the current ICH-GCP guidelines.

Date/Place 20.8.2007 / Bern Signature [illegible]

STUDY MANAGEMENT AND MONITORING

Name:<br>Address:	Christian Burri, PD Dr.<br>SCIH, Swiss Tropical Institute, Socinstrasse 57, CH 4002 Basel
Phone:<br>Fax:	+41 61 225 26 61<br>+41 61 225 26 78

I, the undersigned, have reviewed this Protocol, including Appendices and I will conduct the clinical study as described and will adhere to ICH-GCP and all the ethical and regulatory considerations stated.

Date/Place 17.8.2007 / BASEL Signature [illegible]

STATISTICIAN

Name:<br>Address:	Ali Mohamed Ali<br>Ifakara Health Research and Development Center (IHRDC)<br>Po Box 78 373<br>Dar Es Salaam, Tanzania (Republic of)
Phone:<br>Fax:	+255 22 277 47 56; +255 23 2440065<br>+255 22 277 47 14; +255 23 2440064

I, the undersigned, have reviewed the protocol, including Appendices, and I will conduct the clinical study as described and will adhere to ICH-GCP Guidelines and all the ethical and regulatory considerations stated.

Date/Place__________________________________ Signature____________________________

List of Abbreviations

Ab	Antibody
AE(s)	Adverse Event(s)
ALAT	Alanine Aminotransferase
ASAT	Aspartate Aminotransferase
BDH	Bagamoyo District Hospital
BRTU-IHRDC	Bagamoyo Research and Training Units
CMI	Cell Mediated Immunity
CNS	Central nervous system
CRC	Clinical research center
CRF	Case Report Form
CTCAE	Common Terminology Criteria for Adverse Events
EKBB	Ethikkommission beider Basel
ELISA	Enzyme-linked Immunosorbent Assay
EMEA	European Medicines Evaluation Agency
ENT	Ear Nose Throat
GCP	Good Clinical Practice
HIV	Human immunodeficiency Virus
IB	Investigator's Brochure
ICH	International Conference on Harmonisation
IFA	Immunofluorescence Assay
IgG	Immunoglobulin G
IRB/IEC	Institutional Review Board/Independent Ethics Committee
IRIV	Immunopotentiating Reconstituted Influenza Virosomes
MUAC	Middle Upper Arm Circumference
PMU	Pharmaceutical Medicine Unit
SAE(s)	Serious Adverse Event(s)
SOP	Standard Operating Procedure
STI	Swiss Tropical Institute

Synopsis

**STUDY NUMBER**	**PMAL03**
TITLE OF THE STUDY	A phase Ib double-blind randomized controlled age-deescalating trial of two virosome formulated anti-malaria vaccine components (PEV301T and PEV302T) administered in combination (PEV3B) to healthy semi-immune Tanzanian volunteers
CLINICAL INVESTIGATORS + STUDY CENTER	Drs Blaise Genton & Salim Abdulla<br>Bagamoyo site<br>IHRDC, Tanzania
PLANNED STUDY PERIOD + CLINICAL PHASE	October 2007 to December 2008<br><br>Phase Ib
INDICATION AND RATIONALE	No vaccine exists today against malaria. Virosomes represent an innovative antigen delivery system, which has already proven its suitability to elicit protective immune responses against subunit vaccine components in humans. The aim of this trial is to proof the concept that virosomes are a suitable delivery system for malaria peptides in populations living in endemic areas, in particular that they allow boosting of pre-existing immunity. This will be investigated with two prototype synthetic *P. falciparum* malaria vaccine components. More than 45 million virosome-based vaccine units have been applied so far in humans, including infants in developing countries, proving that virosomes induce a fast and very specific immune response and are very well tolerated.
OBJECTIVES	Primary<br>- To demonstrate the safety and tolerability of the combination of two virosome formulated malaria peptidomimetics in malaria semi-immune subjects<br>Secondary<br>- To determine the humoral and cellular immune response against two virosome formulated malaria peptidomimetics
ENDPOINTS	Safety and Tolerability:<br>• Occurrence of local and systemic adverse events<br>• Occurrence of clinically significant hematological and biochemical abnormalities<br><br>Immunogenicity:<br>• ELISA for antibody titers against PEV301T and PEV302T, performed by Pevion Biotech Ltd.<br>• Western Blotting and IFA for antibody titers crossreactive with *P. falciparum* parasites (blood stages and sporozoites, respectively), performed by STI<br><br>Ancillary:<br>• Parasite growth/invasion inhibition assays, performed at STI<br>• IgG isotyping, performed at STI<br>• Influenza ELISA/HIT, performed at Pevion Biotech Ltd.<br>• CMI performed at IHRDC and STI

**METHODOLOGY**		
DESIGN		Single centre, randomized, controlled, double-blind, age-deescalating parallel groups
SUBJECTS	NUMBER	A total number of 50 volunteers, 10 adult males and 40 children of both sexes
	POPULATION	Healthy volunteers
	INCLUSION CRITERIA	1. Male volunteers aged between 18 and 45 years for the adult group, and children of both sexes aged 5-9 years for schoolchildren group<br>2. Written informed consent obtained from the volunteer (adult) or guardian/ legal representative (children). In case patient is illiterate, an impartial witness should be present during the entire consent procedure<br>3. Free of obvious health problems as established by medical history and clinical examination before entering the study<br>4. Body Mass Index between 18 and 30 for adults; MUAC >12 for children
	EXCLUSION CRITERIA	1. Use of any investigational or non-registered drug or vaccine within 30 days preceding the first dose of study vaccine, or planned use during the study period and safety follow-up<br>2. Chronic administration (defined as more than 14 days) of

		immunosuppressants or other immune-modifying drugs within six months prior to the first vaccine dose (For corticosteroids, this will mean prednisone, or equivalent, ≥ 0.5 mg/kg/day. Inhaled and topical steroids are allowed.)<br>3. Any chronic drug therapy to be continued during the study period<br>4. Any confirmed or suspected acquired immunosuppressive or immunodeficient condition, including human immunodeficiency virus (HIV) infection, or history of congenital or hereditary immunodeficiency<br>5. History of allergic disease or reactions likely to be exacerbated by any component of the vaccine<br>6. Acute disease at the time of enrolment. Acute disease is defined as the presence of a moderate or severe illness with or without fever (defined as temperature >37.5°C)<br>7. Acute or chronic, clinically significant pulmonary, cardiovascular, hepatic or renal functional abnormality, as determined by physical examination or laboratory screening tests<br>8. Acute or chronic diabetes<br>9. History of chronic alcohol consumption and/or intravenous drug abuse
VACCINE group AV	FORMULATION / DOSE	PEV3B (PEV301T (50 µg AMA49-C1) plus 302T (10 µg UK39))
VACCINE group CV		PEV3B (PEV301T (50 µg AMA49-C1) plus 302T (10 µg UK39))
COMPARATOR group AP		Inflexal V
COMPARATOR group CP		Inflexal V
VACCINATION	ROUTE OF ADMINISTRATION	i.m. in M. deltoideus, 1st left, 2nd right hand side
	DURATION AND FREQUENCY	A total of two injections at study days 0, and 90 (86-94)

CRITERIA FOR EVALUATION

SAFETY AND CMI	• Occurrence of adverse events: assessment of local and systemic reactions 2 hours after each vaccination, at day 1, 2, 3, 7, 14 (±2), 30 (±4) and during a monthly visit until the end of volunteer's study schedule.<br>• Occurrence of clinically significant hematological and biochemical abnormalities (hematological and biochemical analysis will be carried out at baseline by the subject, days -10 to -2), and on days 7 (±2), 90 (±4) and 97 (±4)
TIMING of SPECIMEN SAMPLING:<br>for HUMORAL RESPONSE	At baseline (days -10 to -2, at day 30 (±4), 90 (±4) (day of the 2nd vaccination), 120 (±4), 180 (±7) and 365 (±14).
For CMI	Cell mediated immune responses will be assessed at day of vaccination (day 0), two weeks after second vaccination (on day 104(±4) and one year after first vaccination on day 365 (±14).
EVALUATION CRITERIA	Increase of the antibody titers both against the vaccine components and *P. falciparum* parasites
TESTS PERFORMED	See Immunogenicity & Ancillary endpoints

PROCEDURE	Volunteers will be screened, enrolled, injected with the vaccine or comparator and followed by the clinicians at the Bagamoyo site.<br>First, 10 adult males will be enrolled and randomized in 2 groups: Group AV (n=8) will be injected with the **vaccine combination (PEV3B)** and group AP (n=2) will be vaccinated with the **comparator**. 5 weeks later, 8 children will be enrolled first and randomized in 2 groups: Group CV (n=6) will be injected with the **vaccine combination (PEV3B)** and group CP (n=2) will be vaccinated with **comparator**. 1 week later, the rest of the cohort (n=32) will be enrolled and randomized in 2 groups: Group CV (n=26) will be injected with the **vaccine combination (PEV3B)** and group CP (n=6) will be vaccinated with **comparator**.<br><br>The pre-vaccination period (days -10 to -2) covers the following consecutive procedures: (1) screening of subject (eligibility), (2) written informed consent by healthy volunteer, (3) baseline examination and blood sampling, (4) randomization to either the **vaccine combination (PEV3B), PEV301T** 50 µg **plus PEV302T** 10 µg, or to **Inflexal (comparator)**.

	The first treatment with either the vaccine or the comparator represents day 0 of the subject's study schedule.<br><br>After each injection, the volunteers have to remain in the clinic for 2 hours for the assessment of local and systemic reactions.<br><br>**Immunogenicity** assessments for **humoral immune response** will be made at baseline (days -10 to -2), day 30 (±4), day 90 (±4) (day of 2nd vaccination), 120 (±4), 180 (±7), and 365 (±14).<br><br>**Cellular immune responses** will be assessed before 1st vaccination (day 0), two weeks after 2nd vaccination (day 104 ±2), and one year after the 1st vaccination (day 365 ±14)<br><br>**Safety assessments** will be made by the investigator at baseline (days -10 to -2, before the 1st immunization) and at day 1, 2, 3, 7, 14, 30 after each vaccination.<br><br>**Standard blood chemistry and hematology** will be assessed at baseline (days -10 to -2), and at day 7 (±2), 90 (±4) and 97 (±4).<br><br>**Parasitological density** will be assessed at screening, on days 7, 90 (+4) and 97 (+4**)**
STATISTICAL METHODS AND EVALUATION OF DATA	<u>Demographic data</u> of each study group will be tabulated.<br><u>Safety data:</u> Listings will be made of the safety data collected at each time point.<br>Descriptive statistics will be used to analyze adverse events (AEs) including intercurrent illnesses. The numbers of AEs and their severity will be reported using frequency tables.<br><u>Immunogenicity data</u>: Immunological data for each time point will be analyzed separately.<br>(i) Descriptive statistics (minimum, maximum, median, geometric mean, arithmetic mean and quartiles) will be computed for each immunological measure and each time point, separately for the 4 groups (AV, AP, CV, CP).<br>(ii) For each volunteer, the ratio of the immunological measure to that assessed at baseline (during screening) will be computed. Descriptive statistics of these ratios (minimum, maximum, median, geometric mean, and quartiles) will be computed for each immunological measure and each time point, separately for each group.<br>(iii) Wilcoxon test will be used to compare the immunological measures for each time point in each group.

Schedule of Assessments (Visits)

Study weeks	-1	1	1-2	4-9	13	13-14	18	22-48	52
Study days	-10 to -2	0	1, 2, 3, 7, 14(±2) [a]	30, 60 (±4)	90 (±4)[b]	91, 92, 93, 97, 104 (±4)	120 (±4)	150,180 (±7)[c], 211, 241, 272, 302, 333	365 (±14)[d]
Study period	Screening, Baseline	1st vaccination	Follow-ups		2nd vaccination		Follow-ups		
Examination	Volunteer eligibility, Medical history, in-/exclusion criteria, informed consent	Assessment of health status (pre-vaccination)	Assessment of health status, Recording of AEs	Assessment of health status, Recording of AEs	Assessment of health status (pre-vaccination), Recording of AEs	Assessment of health status, Recording of AEs	Assessment of health status, Recording of AEs	Assessment of health status, Recording of AEs	Assessment of health status, Recording of AEs
**Vaccination**		**0.5 ml i.m. (left)**			**0.5 ml i.m. (right)**				
Blood Sampling: safety (hematology,[1]chemistry[2] and parasitology)	2 ml blood	0.5 mL blood	2 ml blood on day 7 only		2 ml blood	2 ml blood on day 97 only			
Blood Sampling: humoral immunology	5 ml blood (baseline values, incl. Influenza)			3 ml blood on day 30 only	3 ml blood (pre-vaccination)		3 ml blood	3 ml blood on day 180 only	3 ml blood
Blood Sampling: CMI		7 ml children<br>25 ml adults				7 ml children<br>25 ml adults on day 104			7 ml children<br>25 ml adults

[1] hematology includes: hemoglobin, hematocrite, RBC count, WBC count and differential, platelets count

[2] blood chemistry includes ASAT/ALAT, alk. Phosphatase, creatinine, CRP

[a] Must be 1, 2, 3, 7, 14 days after vaccination

[b] Should be 3 months after vaccination

[c] Should be 6 months after the 1st vaccination

[d] Should be 1 year after the 1st vaccination

Flow Chart

Month of the year	Days	Group AV (8) + AP (2)<br>PEV301T/302T or Inflexal V	First<br>Group CV (6), CP (2)<br>PEV301T/302T or Inflexal V	Remaining<br>Group CV (26), CP (6)<br>PEV301T/302T or Inflexal V
~Oct. 2007	-10-(-2)	Screening		
	0	Immuno CMI,<br>**Vaccination 1**		
	7	Safety lab (haemato. bioch.)		
	30	Immuno. Lab (Ab)		
~Nov. 2007	25-33		Screening	
	35		Immuno CMI,<br>**Vaccination 1**	
	32-40			Screening
	42		Safety lab (haemato. bioch.)	Immuno CMI,<br>**Vaccination 1**
	49			Safety lab (haemato. bioch.)
~Dec. 2007	65		Immuno. Lab (Ab)	
	72			Immuno. Lab (Ab)
~Jan. 2008	90	Ab + **Vaccination 2**		
	97	Safety lab<br>(haemato. bioch.)		
	104	CMI lab		
~Feb. 2008	120	Ab lab		
	125		Ab + **Vaccination 2**	
	132		Safety lab (haemato. bioch.)	Ab + **Vaccination 2**
	139		CMI lab	Safety lab (haemato. bioch.)
	146			CMI lab
	155		Ab lab	
	162			Ab lab
~April 2008	180	Ab lab		
	215		Ab lab	
	222			Ab lab
~Oct. 2008	365	Ab+ CMI		
~Nov. 2008	400		Ab+ CMI	
	407			Ab+ CMI
~Dec. 2008		**End of field work**		

Table of Contents

1 Introduction 14
1.1 Background 14
1.2 Virosomes 14
1.3 Malaria antigens 14
1.4 Clinical trials in humans 15
1.4.1 Phase Ia in Switzerland 15
1.4.2 Phase IIa in United Kingdom 16
1.5 Rationale 16
2 Objectives 17
2.1 Primary objective 17
2.2 Secondary objective 17
3 Study Design 18
4 Study Population 18
4.1 Number of subjects 18
4.2 Inclusion criteria 18
4.3 Exclusion criteria 18
4.4 Contraindications to repeated vaccination/termination of vaccination 19
5 Investigational Plan 19
5.1 General study aspects 19
5.2 Detailed description of study stages/visits 20
5.2.1 Screening of potential study participants 20
5.2.2 Vaccinations 20
5.2.3 Follow-up visits 20
5.3 Blood sampling 20
5.3.1 Safety 20
5.3.2 Immunology 21
5.3.3 Parasitology 21
5.4 Concomitant medication/treatment 21
5.5 Subject withdrawals / dropouts / lost to follow-up 22
6 Study Vaccine(s) and Administration 22
6.1 Origin of vaccine(s) 22
6.2 Investigational Product 22
6.3 Treatment allocation and randomization 23
6.4 Dosage and administration 23
6.5 Storage 23
6.6 Method of blinding and unblinding 24
6.7 Vaccine accountability 24
6.8 Concomitant medication/treatment 24
7 Health Economics 24
8 Adverse Events (AEs) 24
8.1 Definitions 24
8.2 Surveillance period for occurrence of adverse events 26
8.3 Recording adverse events 26
8.3.1 Solicited adverse events 26
8.3.2 Unsolicited adverse events 27
8.4 Assessment of intensity 27
8.5 Assessment of causality 27

8.6 Follow-up of adverse events and assessment of outcome 28
8.7 Reporting of adverse events 28
8.8 Reporting of serious adverse events 28
8.9 Follow-up of serious adverse events 29
8.10 Pregnancy 30
8.11 Treatment of adverse events 30
9 Study Completion and Discontinuation 30
9.1 Definitions 30
9.2 Reasons for drop-out 30
9.3 Procedures for handling subjects discontinued 30
9.4 Data and Safety Monitoring Board 30
10 Data evaluation 31
10.1 Data handling 31
10.2 Primary tests 31
10.3 Secondary tests 31
10.4 Statistical hypothesis 31
10.5 Sample size calculation 31
10.6 Statistical analysis 32
10.7 Final analysis 32
10.7.1 Safety evaluation 32
10.7.2 Immunogenicity evaluation 32
10.8 Interim analysis 33
10.9 Criteria for inclusion in the analysis 33
11 Ethics and regulatory considerations 33
11.1 Independent Ethics Committee (IEC) / Institutional Review Board (IRB) 33
11.2 Informed consent 33
11.3 Benefits and Risks 34
12 Administrative Matters 34
12.1 Protocol and Investigators Brochure (IB) 34
12.2 Confidentiality 35
12.3 Documentation and Material Supplies 35
12.4 Financial compensation to the participants 35
12.5 Monitoring 35
12.6 Source Documents and Case Report Forms (CRFs) 35
12.7 Quality assurance audit 35
12.8 Compensation for medicine-induced injury 36
12.9 Early termination 36
12.10 Publication of data and protection of trade secrets 37
12.11 Intellectual property rights 37
12.12 Entry violations 37
13 References 37
Appendix 1: Helsinki Declaration xxxix

1 Introduction

1.1 Background

It is assumed that vaccines have prevented more infectious diseases than any other medical intervention, except sanitation. With currently up to 300 million affected people, malaria continues to be one of the major burdens on public health in many tropical countries. Because of the spread of drug-resistant parasites and the appearance of insecticide-resistant mosquitoes, the development of an effective vaccine against the most severe form of malaria caused by *Plasmodium falciparum* is an urgent priority.

Since cultivation of malaria parasites on a sufficiently large scale has turned out to be difficult for mass vaccination, efforts in malaria vaccinology are currently focused on the development of subunit vaccines. Although a number of vaccine candidate antigens have been identified, clinical trials with first vaccine formulations including recombinant viruses, DNA vaccines and adjuvanted recombinant malaria proteins, fusion proteins and synthetic peptides, have met only limited success. Both RTS,S in Mozambique and Combination B in Papua New Guinea have shown promising results, but both of them will need further field assessment and refinement, before commercialization is envisagable. Innovative approaches and new technology platforms are urgently required in order to improve the safety and immunogenicity of vaccines. The proposed product and technology development project represents a highly innovative approach for subunit vaccine design, and the concept has been proven in a Phase I human trial in non-immune Caucasian volunteers.

1.2 Virosomes

There is mounting evidence that the majority of natural anti-malarial immune responses (and accordingly responses to entire recombinant malaria proteins) are useless or even counterproductive. Therefore our approach is, to focus the immune response to selected protective epitopes of key malaria antigens.

We have developed new approaches and technologies for the design of epitope-focussed vaccines [1-6]. Starting point was to investigate the possibility of associating peptide or protein antigens in a defined fashion using immunopotentiating reconstituted influenza virosomes (IRIV). These are spherical, unilammelar vesicles, prepared by detergent removal from a mixture of natural and synthetic phospholipids and influenza surface glycoproteins. They have been shown to be a highly effective means of enhancing the immune response to a variety of antigens. The haemagglutinin membrane glycoprotein of the influenza virus plays a key role in the activity of IRIV. This major antigen of influenza virus is a fusion-inducing component, which facilitates antigen delivery to immunocompetent cells. In the hepatitis A vaccine Epaxal, the first licensed vaccine in which IRIV are used as a delivery system for a non-influenza antigen, the hepatitis A antigen spontaneously binds to the IRIV. We have developed and evaluated a method to couple antigens to phosphatidylethanolamine and to integrate the phospholipid-antigen conjugates into the virosome membrane during the virosome reconstitution process. In experiments in mice, the presentation of multiple copies of the antigen on the virosome surface induced strong antibody responses.

1.3 Malaria antigens

The next step was to couple peptides of surface loops of malaria antigens to the surface of the virosomes. Due to their inherent flexibility, linear peptides often elicit antibodies that bind to denatured proteins, but fail to recognize the same sequences in native protein structures. This is one problem that has so far hindered the application of synthetic peptides in vaccine design. Other problems are that peptides in serum have only a limited stability against proteolysis, and that their immunogenicity when administered as conjugates in human-compatible adjuvants is often weak. We have now shown that these problems may be alleviated by using conformationally defined peptidomimetics coupled to virosomes. We have

identified and optimized conformationally restricted cyclic peptide structures that mimic surface loops of two key malaria vaccine candidate antigens; the NPNA repeat region of circumsporozoite protein (CSP) and the apical membrane antigen-1 (AMA-1).
Starting with a small cyclic peptide containing portions of the NPNA-repeat region of CSP we have identified and profiled an optimized peptide, which very efficiently elicits antibodies which cross-react with *P. falciparum* sporozoites and inhibit sporozoite invasion of human liver cells. These results demonstrated that it is possible to start from a lead structure and design a compound with optimal immunological properties in a stepwise process. With AMA-1, we used two alternative approaches to identify a lead structure for peptide development. The first approach, immunological studies with a library of synthetic template-bound cyclic 12-mer peptidomimetics, turned out to be less successful than a second strategy in which a larger synthetic peptide structure was used as a starting point. Some of the monoclonal antibodies (mAbs) generated against this structure had growth-inhibitory activity against blood stage parasites *in vitro*.
We compared a peptide-virosome formulation with the same peptide presented as a multiple antigenic peptide (MAP) construct, in which several copies of the peptide are adsorbed to an alum-adjuvant. Both formulations elicited comparable levels of anti-peptide antibody responses in mice. However, only the antibodies against the virosome formulation bound to the parasites. This indicated that phosphatidylethanolamine-coupled antigens were located on the surface of the virosomes without their conformation being disturbed, whereas adsorption to alum dramatically disturbed their conformation.
Our joint preclinical research had identified the two virosome products PEV301T [incorporating an Apical Membrane Antigen-1 (AMA-1) peptide] and PEV302T [incorporating a Circumsporozoite Protein peptide (CSP)], which we regarded as suitable components of a virosome malaria vaccine.

1.4 Clinical trials in humans

1.4.1 Phase Ia in Switzerland

Proof of the concept was demonstrated in a Phase Ia clinical trial conducted in non-immune Caucasian volunteers in Switzerland from 2003-2005 (Genton et al, in press) [7]. The design was a single blind, randomized, controlled, dose-escalating study involving 46 healthy Caucasian volunteers aged 18-45 years. Five groups of 8 subjects received virosomal formulations containing 10 µg or 50 µg of AMA 49-C1, the apical membrane antigen-1 (AMA-1) derived synthetic phospatidylethanolamine (PE)-peptide conjugate or 10 µg or 50 µg of UK39, the circumsporozoite protein (CSP) derived synthetic PE-peptide conjugate or a mixture of both virosomal vaccine components with 50 µg of both antigens each. A control group of 6 subjects received unmodified virosomes. Injections were done on days 0, 60 and 180. In terms of safety, the vaccine was very well tolerated. No serious or severe adverse events (AEs) related to the vaccine occurred. Local adverse events were only pain (no redness, no swelling). Generally, no difference in the distribution of the systemic AEs between either the doses applied (10 respectively 50 µg) or the synthetic antigen vaccines (PEV301 and PEV302) used for immunization was found. In terms of immunogenicity, both PEV301 and PEV302 elicited already after two injections a synthetic peptide-specific antibody response measured by ELISA in all volunteers immunized with the appropriate dose. In the case of PEV301 the 50 µg antigen dose was associated with a higher mean antibody titer and seroconversion rate than the 10 µg dose. In contrast, for PEV302 mean titer and seroconversion rate were higher with the lower dose. Combined delivery of PEV301 and PEV302 did not interfere with the development of an antibody response to either of the two antigens. Vaccination with the conformationally constrained peptide derived from the NANP repeat region of CSP also induced parasite-binding antibodies measured by IFA, which inhibited sporozoite migration and invasion in a dose-dependent manner [8]. No relevant antibody responses against the two malaria antigens were observed in the control group receiving unmodified virosomes.

1.4.2 Phase IIa in United Kingdom

Following the Phase I trial, a Phase IIa artificial challenge trial was conducted in non-immune Caucasian volunteers in 2006 to investigate potential protection against *P. falciparum* parasitaemia. In this study, we observed evidence of blood stage protection for the first time in humans, although there was no sterile protection. One PEV3A vaccinated volunteer was diagnosed late, on day 20 (compare mean day of diagnosis in unvaccinated control volunteers of 11.8 days) and morphologically abnormal parasites (crisis form) were present in the blood of all PEV3A vaccinated volunteers, and in only 2/6 controls (p=0.001). PCR results from PEV3A vaccinated volunteers show early control of parasitaemia by some volunteers. The mean parasite growth rate was lower in volunteers vaccinated with PEV3A compared to unvacinated volunteers.
This study confirmed the safety and immunogenicity data from the Phase Ia trial, and showed that the artificial challenge boosted vaccine-induced immune responses (IFA and Western Blotting). The longer duration of time up to parasitological diagnosis in the vaccine group may suggest a reduction of parasite leaving the liver.

1.5 Rationale

No vaccine exists today against malaria. Attempts to produce a multi-stage subunit vaccine against the malaria parasite *Plasmodium falciparum* have so far met with limited success. Cumulated experience with the clinical profiling of previous malaria vaccine candidates indicates that new strategies both for the targeting of the immune response to suitable antigenic determinants of the parasite and for the safe and appropriate delivery of antigens are required. We therefore have developed an alternative approach for the design of a malaria vaccine, which is based on the delivery of peptidomimetics by immunostimulating influenza virosomes (IRIV's).
IRIV represent an adjuvant carrier system that is already incorporated in two vaccines registered for human use (EU and Canada). More than 25 million IRIV-based vaccine units have been applied so far, proving that the virosomes induce a fast and very specific immune response and are very well tolerated. Moreover, thousands of infants have already received the virosome-formulated vaccines, mainly the virosomal hepatitis A vaccine Epaxal. This clinical experience has proved to be safe in this age group, which is a major advance for the development of malaria vaccines that are targetted primarily to this age group. Our pre-clinical and clinical research has demonstrated that it is possible to induce malaria parasite growth and invasion inhibitory antibodies by delivering synthetic peptidomimetics of crucial protein surface loops of different parasite development stages on the surface of IRIV to the immune system.
Sequential rounds of peptidomimetic optimization have lead to the definition of two candidate components (PEV301 and PEV302) for a virosomal multi-stage malaria vaccine. In experimental animals and humans, these induce highly effective antibody responses against the merozoite and the sporozoite stages of the parasite, respectively.
The ultimate goal is to design a multistage IRIV-based malaria vaccine that would include at least 4 peptides (AMA-1, CSP, MSP1 and probably MSP3 or SERA. This vaccine should go in Phase IIb efficacy trial in Africa. Prior to that stage, we would like to conduct of proof of concept Phase Ib trial in a semi-immune population of adults and children, in order to demonstrate the safety and immunogenicity of our two-component vaccine candidate. We would like in particular to investigate the magnitude of the boosting (if any) of naturally acquired antibody responses (pre-existing immunity) in individuals living in endemic area. The proposed study should justify and speed up the development and testing of the four-component multistage vaccine. The lack of complete understanding of the immune processes for malaria and good correlates of protection in humans necessitates the conduct of phase I and phase II studies in endemic populations to properly assess the potential of a candidate vaccine.

The slightly longer duration of mean time to diagnosis and the delay of parasitaemia occurrence in one volunteer in the Phase IIa trial provide only limited insight to the potential of this vaccine. Because of that, and because there is up to now no formal demonstration that proof of efficacy (protection) in a Phase IIa translates into efficacy (protection) in targets groups in endemic areas, we believe it is justified to perform a proof of concept study (safety and immunogenicity) in a limited number of volunteers, including children, in the field. For the same reasons, we believe that it is premature at this stage to delete the CSP component, on the sole basis of low efficacy in the Phase Ia trial. As a next step of optimization we further improved the virosomal malaria vaccine candidates by modifications allowing the lyophilization of the preparations (PEV301T and PEV302T) without changing their immunogenic properties.

This protocol proposes to assess the potential of two candidate vaccine components (PEV301T and PEV302T) in endemic populations as the early stage of a development of a virosomal multi-stage malaria vaccine. Outside assessing the safety of the tested vacine, this trial aims at evaluating its immunogenicity, in terms of humoral and cellular responses. T cells are supposed indeed to play a critical role in protection against malaria. CD8 T cells mediate protection against the liver stages while CD4 T cell have been associated with protection against both liver and blood stages. Additionally, CD4+CD25+ regulatory T cells have been implicated in the suppression of malaria-induced immunopathology. Even in the case of vaccine components that achieve protection primarily through the generation of parasite-inhibitory antibodies, T cells are of crucial importance for the development of immunological memory and for the boosting of antibody responses through natural challenge. An effective malaria vaccine therefore requires optimal function of the different T cell subsets. It will be of great importance for the rational design and optimisation of malaria vaccine candidates to come to a better understanding of the immune responses elicited and to eventually develop surrogate markers of protection also for cellular immune responses. This is why we propose to characterize vaccine-specific T cell responses in volunteers living in a malaria-endemic setting and to investigate whether children who develop clinical malaria after vaccination show distinct signatures of vaccine-specific circulating T cells, with a particular emphasis on the number of regulatory and memory T cells and their respective cytokines produced.

The comparator vaccine chosen is the influenza vaccine Inflexal V® (Berna Biotech, Bern, Switzerland). This choice is based on epidemiological and scientific considerations. There is indeed ample documentation of the circulation of influenza viruses in Africa, including the strains that are comprised in Inflexal V®. In Tanzania, a study showed that women had an influenza antibody status that was similar to the one found in the Netherlands (Masurel 1987) [9]. Also, WHO is urging to consider seriously the problem of influenza in Africa because of the potential high morbidity linked to this virus (http://www.who.int/mediacentre/news/releases/2003/pr13/en/). The adults and children of the comparator group should therefore clearly benefit from this vaccine. In addition, this comparator has the advantage of including the same constituant (adjuvant) as the tested vaccine. Indeed, Inflexal V® is also formulated in virosomes, which allows to have most precise assessment of the contribution of the malaria antigen to the safety and immunogenicity data.

2 Objectives

2.1 Primary objective

- To demonstrate the safety and tolerability of the combination of two virosome formulated malaria peptidomimetics in malaria semi-immune subjects

2.2 Secondary objective

To determine the humoral and cell mediated immune response against two virosome formulated malaria peptidomimetics given in combination, in particular the boosting effect on naturally acquired immune responses.

3 Study Design

This prospective phase I, single centre, randomized, double blind, controlled study will be conducted in 50 healthy volunteers. Eligible study participants will be block-randomized into four groups: Adult vaccine group (AV) receiving PEV301T 50 µg plus 302T 10 µg (n = 8), adult group receiving comparator (AP) (n=2), children vaccine group receiving PEV3B (PEV301T (50 µg AMA49-C1) plus 302T (10 µg UK39)) (CV) (n = 32), and children group receiving comparator (CP) (n=8).

Study participants will be vaccinated twice, i.e. at days 0, and 90 (±4) and followed-up daily for 3 days after each vaccination.

Groups CV & CP will be started 5 weeks after the 1st vaccination of groups AC and AP. In addition, the CV and CP groups will be split in two, the first 6 CV and 2 CP will be vaccinated first, and one week later the remaining 26 CV and 6 CP if there is no safety concerns in the first lot.

The comparator groups will be distributed and serve as a control in all groups receiving the active product.

The total duration of the study for each participant is one year, and the number of visits is 24

4 Study Population

4.1 Number of subjects

A total of 50 healthy subjects will be enrolled into the study, 10 male adults and 40 children of both sexes.

Only subjects for whom the investigator believes the requirements of the protocol will be complied with (e.g. return for follow-up visits) should be enrolled in the study.

4.2 Inclusion criteria

The following criteria should be checked at the time of study entry (screening visit). If any does not apply at the time of study entry, the subject must not be included in the study:

1. Male volunteers aged between 18 and 45 years for the adult group, and children of both sexes aged 5-9 years for schoolchildren group
2. Written informed consent obtained from the volunteer (adult) or guardian/ legal representative (children). In case patient is illiterate, an impartial witness should be present during the entire consent procedure
3. Free of obvious health problems as established by medical history and clinical examination before entering the study
4. Body Mass Index between 18 and 30 for adults; MUAC >12 for children

4.3 Exclusion criteria

The following criteria should be checked at the time of study entry. If any apply at the time of study entry, the subject must not be included in the study:

1. Use of any investigational or non-registered drug or vaccine within 30 days preceding the first dose of study vaccine, or planned use during the study period and safety follow-up
2. Chronic administration (defined as more than 14 days) of immunosuppressants or other immune-modifying drugs within six months prior to the first vaccine dose (For

corticosteroids, this will mean prednisone, or equivalent, ≥ 0.5 mg/kg/day. Inhaled and topical steroids are allowed.)
3. Any chronic drug therapy to be continued during the study period
4. Any confirmed or suspected acquired immunosuppressive or immunodeficient condition, including human immunodeficiency virus (HIV) infection, or history of congenital or hereditary immunodeficiency
5. History of allergic disease or reactions likely to be exacerbated by any component of the vaccine
6. Acute disease at the time of enrolment. Acute disease is defined as the presence of a moderate or severe illness with or without fever (defined as temperature >37.5°C)
7. Acute or chronic, clinically significant pulmonary, cardiovascular, hepatic or renal functional abnormality, as determined by physical examination or laboratory screening tests
8. Acute or chronic diabetes
9. History of chronic alcohol consumption and/or intravenous drug abuse

If a subject signs the consent form but withdraws his/her consent after the 1st injection, he/she will abandon the study as of the date of withdrawal. The subject will attest the withdrawal on the consent form and the reason shall be noted at the end of the CRF. Data collected until the date of withdrawal will be used for the statistical analysis of safety.

If a subject is lost to follow-up or drops out, this fact shall be noted at the end of the CRF (definitions see 9.1). Data collected for such subjects will be used for the statistical analysis until the date of lost to follow-up or dropout.

4.4 Contraindications to repeated vaccination/termination of vaccination

The following adverse events constitute absolute contraindications to further administration of PEV3B; if any of these adverse events occur during the study, the subject must not receive additional doses of vaccine but may continue other study procedures at the discretion of the investigator. The subject must be followed until resolution of the event, as with any adverse event (see Section 8):

- Anaphylactic reaction following the administration of vaccine(s).
- Any clinically relevant immunosuppressive or immunodeficient condition

The following adverse events constitute contraindications to administration of PEV3B at that point in time; if any one of these adverse events occurs at the time scheduled for vaccination, the subject may be vaccinated at a later date, within the time window specified in the protocol (see Schedule of Assessments Page 9), or withdrawn at the discretion of the investigator. The subject must be followed as with any adverse event (see Section 8).

- Acute disease at the time of vaccination. Acute disease is defined as the presence of a moderate or severe illness with or without fever. All vaccines can be administered to persons with a minor illness such as mild diarrhea, mild upper respiratory infection with or without low-grade febrile illness, i.e., temperature ≤37.5°C.
- Temperature > 37.5°C at the time of vaccination.

5 Investigational Plan

5.1 General study aspects

The clinical part of the study will be conducted at the Bagamoyo Research and Training Unit of the the Ifakara Health Research and Development Center (BRTU-IHRDC), and within the premises of the Bagamoyo District Hospital (BDH). Drs Blaise Genton and Salim Abdulla will

be the clinical investigators, assisted by other clinicians at the BRTU-IHRDC who have experience with the conduct of other malaria vaccine trials.

It is the investigator's responsibility to ensure that the intervals between visits/contacts are strictly followed. These intervals during the active phase of the study determine a subject's evaluability in the as per protocol analyses.

5.2 Detailed description of study stages/visits

For information on time points of visits, vaccinations & checks refer to schedule of assessments page 9.

5.2.1 Screening of potential study participants

During the Screening Visit (days -10 to -2) the investigator has to check the eligibility of the potential participants for this study (inclusion/exclusion criteria). Volunteers will not be included if any of the exclusion criteria apply. Volunteers who have signed the Informed Consent Form (or guardian instead of signature of children) will undergo complete physical examination (baseline examination), including vitals signs (blood pressure, pulse, temperature) and body systems (cardiovascular, gastro-intestinal, CNS, ENT, respiratory, urogenital, dermatology). Demographic data will be collected, and a complete clinical history will be recorded, and laboratory tests (including HIV and influenza A titer) will be performed. The informed consent process will involve the standard voluntary counseling and testing for HIV using the national programme being implemented at the BDH. In the event that a subject is tested positive for HIV, he/she will be informed and referred for evaluation and treatment in the Government programme.

All clinical samples (including serum samples) are to be collected using exclusively the material provided. The use of other material could result in the exclusion of the subject from analysis. The investigator must ensure that his/her personnel and the laboratory(s) under his/her supervision comply with this requirement.

5.2.2 Vaccinations

Volunteers will be vaccinated on days 0 and 90 (86 – 94).
Prior to each vaccination, complains and abnormal signs will recorded by the clinician to establish the baseline AE rate.

5.2.3 Follow-up visits

For time points see schedule of assessments, page 10.
Study participants have to report to the study site every day for the first 3 days, i.e. on days 1, 2, 3 and then on days 7, 14 and 30 post 1st and 2nd vaccination. The investigator has to record any adverse events in the CRF, and take blood samples for parasitology, safety and immunology checks (see 5.3.4.). In addition, subjects will be followed once a month up till the end of the study

5.3 Blood sampling

Blood samples will be taken by venepuncture in the arm. Whole blood will be centrifuged. Serum will be stored in -80C° freezer before shipment to Switzerland for antibody assessment. White blood cells will be stored in liquid nitrogen before assessment at the BRTU-IHRDC.

5.3.1 Safety

For time points see schedule of assessments, page 10.

The following blood samples have to be taken: 2 ml whole blood for the assessment of hematological, biochemical and parasitology parameters at baseline, and on days 7 (±2), 90 (±4) and 97 (±4). The following analyses will be performed at the BRTU-IHRDC:

- Hematology:

Hemoglobin
Hematocrit
Red Blood Cell count
White Blood Cell count and differential count
Platelet count

- Blood chemistry:

ASAT
ALAT
Alkaline Phosphatase
Creatinine

5.3.2 Immunology

For time points see schedule of assessments, page 10.
5 ml whole blood will be collected at screening, and 3 ml on days 30 (+4), 90 (+4) (day of the 2nd vaccination), 120 (±4), 180 (±7) and 365 (±14) for the analysis of humoral immune responses and parasitology.
7 ml and 25ml of blood will be collected from children and adult volunteers respectively to assess cellular immune responses on the day of vaccination (day 0), two weeks after 2nd vaccination (day 104, ±4), and one year after 1st vaccination (day 365 ±14).

Due to development of sensitive quantitative assays, evaluation of T-cell mediated immunity (CMI) has improved in recent years. However, the cellular mechanisms that have to be investigated are much more complex than in the case of humoral immune responses and even a minimal set of assays still require large numbers of peripheral blood mononuclear cells. Therefore substantially larger volumes of peripheral blood have to be collected for analysis of T cell responses than for serological tests. 3.5 ml will be needed for PBMC stimulation experiments for testing RNA expression profiles, 3.5 ml will be needed for PBMC labelling with CFSE and flow cytometric analysis after stimulation with different antigens. 7 ml from children will be therefore just sufficient to run a single set of experiment with a minimum number of stimulators and markers analysed. 25 ml from adults will allow to 1. reproduce the experiment once, 2. use a slightly broader spectrum of markers and to 3. develop T cell clones from selected volunteers with very good immune responses

ELISA will be performed at BRTU-IHRDC, STI and Pevion Biotech Ltd. for validation, flow cytometry will be performed at BRTU-IHRDC and all other immunological assays at the Swiss Tropical Institute, if not yet established at BRTU-IHRDC. .

5.3.3 Parasitology

For time points see schedule of assessments, page 10.

0.5 ml of blood collected at screening, on days 7, 90 (±4) and 97 (±4), as well as on days where the subject report fever, will be used to assess presence and density of *Plasmodium* parasites by microscopy according to SOP at BRTU-IHRDC.

5.4 Concomitant medication/treatment

At each study visit/contact, the investigator should question the subject about any medication taken.

Any immunosuppressants or other immune-modifying drugs or treatments, any vaccine other than the study vaccine(s) and any antipyretics administered at ANY time during the period starting 30 days prior to the first dose of study vaccine(s) and ending one month (minimum 30 days) after the last dose of study vaccine(s) must be recorded in the CRF with trade name and/or generic name of the medication, medical indication, total daily dose, route of administration, start and end dates of treatment.

Any other concomitant medication administered prophylactically in anticipation of reaction to the vaccination must also be recorded in the CRF with trade name and/or generic name of the medication, total daily dose, route of administration, start and end dates of treatment and coded as `prophylactic'.

5.5 Subject withdrawals / dropouts / lost to follow-up

Withdrawals / dropouts/ lost to follow-ups will NOT be replaced, except if no injection has been administered.

For more information see chapter 9 "Study Completion and Discontinuation"

The subject should be informed of the right to abstain from participation in the study or to withdraw consent to participate at any time without reprisal.

If a subject signs the consent form but withdraws his/her consent after the 1st injection, he/she will abandon the study as of the date of withdrawal. The subject will attest the withdrawal on the consent form and the reason shall be noted at the end of the CRF. Data collected until the date of withdrawal will be used for the statistical analysis of safety.

If a subject is lost to follow-up or drops out, this fact shall be noted at the end of the CRF (definitions see 9.1). Data collected for such subjects will be used for the statistical analysis until the date of lost to follow-up or dropout

6 Study Vaccine(s) and Administration

6.1 Origin of vaccine(s)

All candidate vaccines to be used have been developed and manufactured by Pevion Biotech Ltd.

The Quality Control Standards and Requirements for each candidate vaccine are described in separate release protocols/Certificate of Analysis and the required approvals have been obtained.

6.2 Investigational Product

The virosome-formulated malaria vaccines PEV3B (PEV301T, PEV302T) and vehicle are supplied in vials in a lyophilized form. Reconstitution with a watery solution will be done at point of injection (< 4 hours before).

PEV3B 0.500 mL Lot Nr.: 03PEVXX
PEV301T and PEV302T: Active substances: 50 µg AMA1 mimetic, 10 µg CSP mimetic, respectively.
Excipients: IRIV, PBS pH 7.4

Inflexal V 0.500 mL Lot Nr.: XX
Active substances: 15 µg hemagglutinin of each three viral strains
Excipients: IRIV, 17 µg lecithin, 3,8 mg dehydrated sodium hydrogenophosphate, 0,7 mg potassium dihydrogenophosphate, 2,4 mg sodium chlorate and 0,5 ml of water for injection.

The vaccines will be blistered and packed in boxes labeled with "Drug for investigational use only" (see example below):

PMAL03

PEV3B

50 µg PEV301T, 10 µg PEV302T
Malaria vaccine
Drug for investigational use only
For i.m. injection

Lot: 03PEVXX
Store at +2 – +8°C
Expiry date: MM.JJJJ
Pevion Biotech Ltd.

6.3 Treatment allocation and randomization

Randomization will be computer-generated. The first block of 10 adults (8 vera, 2 controls) will be randomized separately. Then the children will be randomized by block of 8 (6 vera, 2 controls). The investigator will receive envelopes with numbers (1 to 50) corresponding to the sequence of assignment to the study (subject number). This envelope will be forwarded to an independent nurse that will perform the injection. In the envelope, another number (treatment allocation number) corresponding to a number on the syringe to be administered will be provided.

The 10 adults will be first randomized and vaccinated with the combination PEV3B (50 µg PEV301T and 10 µg PEV302T) (group AV, n=8), or comparator (group AP, n=2).
5 weeks later again, the first block of 8 children will be randomized and vaccinated with the combination PEV3B (50 µg PEV301T and 10 µg PEV302T) (group CV, n=6), or comparator (group CP, n=2). One week later, the remaining 32 children will be randomized in 4 blocks of 8 and vaccinated with the combination PEV3B (50 µg PEV301T and 10 µg PEV302T) (group CV, n=24), or comparator (group CP, n=8).

6.4 Dosage and administration

The combination PEV3B will be provided in a lyophylized form into vials. The vials for each group will be provided in individually labeled boxes. The comparator will be provided in its usal form (prefilled syringes).

0.5 mL PEV3B vaccines or comparator should be administered intramuscularly (i.m.) into the deltoid region of the upper arm. The first application is to be given to left, and the second to right arm, respectively. The vaccine should not be injected into the blood vessels. The vaccination site should be disinfected with a skin disinfectant (e.g. 70% alcohol) prior to vaccination. The Investigator should use only the syringes provided by the sponsor.

The subjects will be observed closely for at least 2 hours after each vaccination and vital signs recorded every 15 mins. Appropriate medical treatment will be readily available in case of a rare anaphylactic reaction following the administration of vaccines. Any reactions that occur during this time must be recorded by the Investigator in the CRF.
The subjects will be instructed to contact the investigator immediately should they manifest any signs or symptoms they perceive as serious.

6.5 Storage

It is essential that Pevion Biotech Ltd can be certain that the study vaccines retain their safety and potency for the duration of their assigned shelf life by proper handling and assurance of the cold chain. All vaccines will be stored in a safe and locked place with no access for unauthorized personnel. They will be kept in a refrigerator (+2°C to +8°C) and must not be frozen.

6.6 Method of blinding and unblinding

On the outside of envelopes, the study subject number corresponding to the sequence of assignment to the study (1-50) will be written and given to the subject or guardian on the day of vaccination when inclusion and exclusion criteria have been checked. The subject or guardian will go in another room where an independent pharmacist and/or nurse will open the envelope. In this envelope, another number corresponding to a number on a syringe (treatment allocation number) will be written. The pharmacist and/or nurse will perform the injection with a syringe that will be covered with an aluminum foil. The investigators will not have access to the vaccination room during the vaccination process.

6.7 Vaccine accountability

The investigator has the obligation to account for all the investigational products and will fill in the respective accountability forms provided. After approval from Pevion Biotech Ltd, both used and unused vaccine vials should be kept up until study closure. The destruction may be performed at the study site using locally approved biosafety procedures and documentation.

6.8 Concomitant medication/treatment

At each study visit/contact, the investigator should question the subject about any medication taken.

Any concomitant medication administered prophylactically in anticipation of reaction to the vaccination must also be recorded in the CRF with trade name and/or generic name of the medication, total daily dose, route of administration, start and end dates of treatment and coded as `prophylactic'.

7 Health Economics

Not applicable

8 Adverse Events (AEs)

The recording of adverse events is an important aspect of study documentation. It is the responsibility of the investigator to document all adverse events according to the detailed guidelines set out below.

The subjects will be instructed to contact the investigator immediately should the subject manifest any signs or symptoms they perceive as serious during the period extending from performance of the first study procedure up to study completion.

8.1 Definitions

<u>Adverse event:</u>

An adverse event is any untoward medical occurrence in a patient or clinical trial subject administered a medicinal product and which does not necessarily have to have a causal relationship with this treatment.

This includes any noxious, pathological or unintended change in anatomical, physiological or metabolic functions as indicated by physical signs, symptoms and/or laboratory detected changes occurring in any phase of the clinical study whether associated with the study vaccine, active comparator and whether or not considered vaccination related. This includes an exacerbation of pre-existing conditions or events, intercurrent illnesses, or vaccine or drug interaction. Anticipated day-to-day fluctuations of pre-existing conditions that do not represent a clinically significant exacerbation need not be considered adverse events. Discrete episodes of chronic conditions occurring during a study period should be reported as adverse events in order to assess changes in frequency or severity.

Adverse events should be documented in terms of signs and symptoms observed by the investigator or reported by the subject at each study visit. A medical diagnosis should be added.

Pre-existing conditions or signs and/or symptoms (including any which are not recognized at study entry but are recognized during the study period) present in a subject prior to the start of the study should be recorded in the medical history part of the subject's CRF.

Adverse events, which occur after informed consent is obtained, but prior to vaccination, will be documented in the medical history part of the subject's CRF.

Although not considered as an adverse event, hospitalization for either elective surgery related to a pre-existing condition, which did not increase in severity or frequency following initiation of the study, or for routine clinical procedures (including hospitalization for "social" reasons) that are not the result of an adverse event, must be recorded in the CRF. If the hospitalization arises from a pre-existing condition, or was planned prior to the first vaccination, it should be recorded in the medical history part of the CRF. If it was planned after the first vaccination, it should be recorded in the adverse event page of the CRF. In both cases, it should be recorded as `Hospitalization (Not an adverse event)', and the relationship to vaccination will be checked "No". These adverse events are not considered as SAE.

<u>Serious adverse events</u>
A serious adverse event is any untoward medical occurrence or effect that any dose

- results in death,
- is life threatening*,
- results in persistent or significant disability/incapacity†,
- requires in-patient hospitalization‡ or prolongation of existing hospitalization
- is a congenital anomaly/birth defect in the offspring of a study subject.

Although not considered as ‘serious adverse events', cancers should be reported in the same way as serious adverse events.

* Life threatening - definition: An adverse event is life threatening if the subject was at risk of death at the time of the event; it does not refer to an event, which hypothetically might have caused death if it were more severe.

† Disabling/incapacitating - definition: An adverse event is incapacitating or disabling if the event results in a substantial disruption of the subject's ability to carry out normal life functions. This definition is not intended to include experiences of relatively minor medical significance such as headache, nausea, vomiting, diarrhea, influenza, injection site reactions and accidental trauma (e.g. sprained ankle).

‡ Hospitalization: In general, hospitalization signifies that the subject has been detained (usually involving at least an overnight stay) at the hospital or emergency ward for treatment that would not have been appropriate in the physician's office or out-patient setting.

Hospitalization for either elective surgery related to a pre-existing condition, which did not increase in severity or frequency following initiation of the study or for routine clinical procedures (including hospitalization for "social" reasons) that are not the result of an adverse event need not be considered as adverse events and are therefore not serious adverse events.

¶ Routine Clinical Procedure definition: One, which is defined as a procedure, which may take place during the study period and should not interfere with the study vaccine administration or any of the ongoing protocol specific procedures.

N.B. If anything untoward is reported during an elective procedure, that occurrence must be reported as an adverse event, either `serious' or non-serious according to the usual criteria.

When in doubt as to whether ‘hospitalization’ occurred or was necessary, the adverse event should be considered serious.

8.2 Surveillance period for occurrence of AEs and SAEs

Serious adverse events will be recorded and followed up on a separate SAE Form as of first vaccination; adverse events will be recorded and followed-up on the Adverse Event Form in the CRF as of first vaccination (day 0).

AEs and SAEs will be assessed and followed up until the outcome is resolved, death or lost to follow-up within a maximum of 30 days after the subject’s participation in the study (30 days after successful completion of the study or 30 days after any early termination); if after this 30-days-period the outcome is neither resolved, death, lost to follow-up, the investigator performs a final assessment and refers the subject to an appropriate medical specialist.

8.3 Recording adverse events

At each visit/assessment, all adverse events either observed by the investigator or one of his clinical collaborators or reported by the subject spontaneously or in response to a direct question will be evaluated by the investigator. Adverse events will be recorded in the Adverse Event form within the subjects CRF. The nature of each event, date and time (where appropriate) of onset, outcome, intensity and relationship to vaccination should be established. Details of any symptomatic/corrective treatment should be recorded on the appropriate page of the CRF.

Adverse events already documented in the CRF, i.e. at a previous assessment, and designated as ‘ongoing’ should be reviewed at subsequent visits, as necessary. If these have resolved, the documentation in the CRF should be completed. If an adverse event changes significantly in frequency or intensity during a study period, a new record of the event will be started.

During a 4-day period after each vaccination adverse events are catagorized into two groups: (1) solicited adverse events and (2) unsolicited adverse events.

8.3.1 Solicited adverse events

Solicited adverse events are predefined adverse events listed in the CRF. Patients are explicitely questionned by the investigator if any of the listed solicited adverse events have occurred. Solicited adverse events, are recorded within a 4-day period immediately after each vaccination.

8.3.2 Local (injection site) solicited adverse events

The following solicited adverse events have been reported in similar studies:

- Pain
- Redness
- Swelling

Solicited local adverse events are considered "vaccine-related".

8.3.3 General solicited adverse events

General solicited adverse events include those observed in the Phase Ia trial and those that have been reported in similar studies with other vaccines:

- Headache (~5% in Phase Ia)
- Fatigue (~2% in Phase Ia)
- Vertigo (~2% in Phase Ia)
- Elevated temperature (>37.5℃) (<1% in Phase Ia)

As a consistent method of soliciting adverse events, first the subject will be asked about the presence or absence of specific signs as listed in sections 8.3.2 and 8.3.3, and then the subject should be asked a non-leading question such as:

"Have you felt different since receiving the vaccine or since the previous visit?"

8.3.4 Unsolicited adverse events

During the 4-day period after each vaccination unsolicited adverse events are recorded on the AE page in the CRF. Unsolicited adverse events are reported spontaneously by the subject or are detected by the investigator.

8.4 Assessment of intensity

Intensity of the following <u>local adverse events</u> should be assessed as described:

Adverse Event	intensity grade	Parameter
Pain	0	Absent
	1	Painful on touch
	2	Painful when limb is moved
	3	Spontaneously painful
Redness	0	Absent
	1	>5 mm
	2	>20 mm
	3	>50 mm
Swelling	0	Absent
	1	>5 mm
	2	>20 mm
	3	>50 mm

For all other adverse events, grading and reporting has to be done according to Common Terminology Criteria for Adverse Events v3.0 (CTCAE).

8.5 Assessment of causality

Every effort should be made by the investigator to explain each adverse event and assess its causal relationship, if any, to administration of the study vaccine(s).

The degree of certainty with which an adverse event can be attributed to administration of the study vaccine(s) (or alternative causes, e.g. natural history of the underlying diseases, concomitant therapy, etc.) will be determined by how well the event can be understood in terms of one or more of the following:

- Reaction of similar nature having previously been observed with this type of vaccine and/or formulation.

- The event having often been reported in literature for similar types of vaccines.

- The event being temporally associated with vaccination or reproduced on re-vaccination.

All solicited reactions will be considered related to vaccination. Causality of all other adverse events should be assessed by the investigator using the following method:

In your opinion, is there a reasonable possibility that the adverse event may have been caused by the study vaccine(s)?

Related	suspicion that there is a relationship between vaccine and AE (without determining the extent of probability); there is a reasonable possibility that the vaccine contributed to the AE
Probable	AE occurs within a reasonable time after the administration of the vaccination and cannot be reasonably explained by other factors (i.e. clinical condition, environmental / toxic factors or other treatments)
Possible	AE occurs within a reasonable time after the administration of the vaccine but can also be reasonably explained by other factors (as mentioned above)
Unlikely	AE does not occur within a reasonable time after the administration of the vaccine (unusual time frame) and can also be reasonably explained by other factors (as mentioned above)
Unrelated	there is no suspicion that there is a relationship between vaccine and adverse event, there are other more likely causes and administration of the study vaccine is not suspected to have contributed to the AE

8.6 Follow-up of adverse events and assessment of outcome

Investigators should follow-up adverse events until the end of the study.

Outcome should be assessed as:
1 = Resolved
2 = Resolved with sequelae
3 = Ongoing at subject study conclusion
4 = Died
5 = Lost to follow up

8.7 Reporting of adverse events

All adverse events occurring during the subject's participation in the study will be assessed, recorded in the CRF and followed-up.

At each visit/assessment, all adverse events, either observed by the investigator or one of his clinical collaborators or reported by the subject spontaneously or in response to a direct question will be evaluated by the investigator. Adverse events not previously documented in the study will be recorded in the AE form within the subject's CRF. The nature of each event, date and time (where appropriate) of onset, outcome, intensity and relationship to vaccination should be established. Details of any corrective treatment should be recorded on the appropriate page of the CRF.

8.8 Reporting of serious adverse events

All Serious Adverse Events (SAEs) must be reported immediately by the investigator without filtration, whether considered to be associated with the study vaccine, active comparator and

whether or not considered vaccination related. The investigator must report SAEs within one calendar day of becoming aware of the event by telephone, telefax or e-mail (if appropriate) to the Study Contact for Reporting Serious Adverse Events as described below. This initial notification should include minimal, but sufficient information to permit identification of the reporter, the subject, the study vaccine, adverse events, and date of onset. The investigator should not wait for additional information to fully document the event before notifying. The responsible recipient will confirm this first notification. The report is then to be followed by submission of a completed SAE Report Form provided by STI/PMU as soon as possible but at latest within 3 calendar days of the initial telephone / telefax or e-mail report detailing relevant aspects of the adverse events in question. All actions taken by the investigator and the outcome of the event must also be reported immediately. For documentation of the SAE and, any actions taken, for outcome and follow-up reports the SAE Report Forms are to be used. Where applicable, hospital case records and autopsy reports should be obtained.

Investigators must report SAEs to the respective ethics committees.

**Study Contact for Reporting Serious Adverse Events**<br>**24/24 hour and 7/7 days availability**
**Office hours:**<br>Name, address: Dr. PD Christian Burri, Swiss Tropical Institute<br>Tel: +41 61 225 26 61<br>Fax: +41 61 225 26 78<br>e-mail: christian.burri@unibas.ch<br><br>Name, address: Dr. Hermann Garden, Swiss Tropical Institute<br>Tel: +41 61 225 26 66<br>Fax: +41 61 225 26 78<br>e-mail: hermann.garden@unibas.ch<br><br>**Outside office hours (nights, weekends):**<br>Doctor of the STI on Call<br>Tel: +41 61 284 81 44
**Back-up Study Contact for Reporting Serious Adverse Events**
Name: Peter Klein, Pevion Biotech Ltd. AG<br>Tel: +41 31 980 62 12<br>Fax: +41 31 980 66 18<br>Mobile phone: +41 79 332 61 44<br>e-mail: peter.klein@pevion.ch

8.9 Follow-up of serious adverse events

SAEs will be followed up until the outcome is a) resolved, b) death, c) lost to follow-up within a maximum of 30 days after the vaccinee's study completion on day 365 ($\pm$14) or 30 days after any early termination. If after this 30 day-period the outcome is neither resolved, death, lost to follow-up, the investigator performs a final assessment and refers the vacinee to an appropriate medical specialist
All follow-up activities have to be reported, if necessary on one or more consecutive SAE report forms in a timely manner. All fields with additional or changed information must be completed and the report form should be forwarded to the Study Contact for Reporting Serious Adverse Events as soon as possible but latest within 5 calendar days after receipt of the new information.
Clinically significant laboratory abnormalities will be followed up until they have returned to normal, or a satisfactory explanation has been provided. Reports relative to the subsequent course of an adverse event noted for any subject must be submitted to the Sponsor.

8.10 Pregnancy

NA.

8.11 Treatment of adverse events

Treatment of any adverse event is at the sole discretion of the investigator and according to current Good Medical Practice. The applied measures should be recorded in the CRF of the subject.

9 Study Completion and Discontinuation

9.1 Definitions

Discontinuation from the study can occur under the following circumstances:

Screening errors:
From an analysis perspective, a 'screening error' is any subject who was enrolled into the study, i.e. was attributed a CRF with subject number, but was withdrawn (before or after randomization) prior to vaccination for reasons such as protocol violation (inclusion/exclusion criteria), 'force majeure' or withdrawal of consent.

Lost to follow-up:
From an analysis perspective, a 'lost to follow-up' is any subject who completed all protocol specific procedures up and including to Day 0, including vaccination, but was then lost to any further follow-up, (no safety information and no efficacy endpoint data ever became available)

Drop-outs:
Any subject for whom data is available for at least one follow-up visit but did not complete the study as foreseen in the protocol.

9.2 Reasons for drop-out

It should be specified on the End of Study page of the CRF, which of the following possible reasons were responsible for drop-out of the subject from the study:

- Adverse events
- Protocol violation (specify)
- Consent withdrawal, not due to an adverse event ,
- Migrated/moved from the study area
- Other (specify)

9.3 Procedures for handling subjects discontinued

Investigators should make an attempt to contact those subjects who do not return for scheduled visits or follow-up. Information gathered should be described on the Study Conclusion page of the CRF and on Medication/Adverse event forms.

9.4 Data and Safety Monitoring Board

A Data and Safety Monitoring Board (DSMB) has been established and includes one independent local monitor (MD clinician, Prof. Karim Manji, Dar Es Salaam, Tanzania), one independent clinician (MD, Dr Mohammed Bakari, Dar Es Salaam, Tanzania), and one

statistician/epidemiologist (Dr Ivo Müller, Papua New Guinea Institute of Medical Research, MAD511, Madang, Papua New Guinea).
The DSMB will be activated, i.e. asked to review, analyse the data and decide on the continuation of the study, if one of the following situations occurs:

- One or more subjects experienced a serious adverse reaction assessed as related to the vaccine by the investigator
- One or more subjects experienced anaphylaxis
- Two or more subjects experienced a severe adverse event not explained by a diagnosis unrelated to vaccination. (For grading of severity see Appendix 4)

- Data evaluationunrelated to vaccination. (For grading of severity see Appendix 4)

10 Data evaluation

10.1 Data handling

The data collected with the CRFs will be entered by data clerks at BRTU-IHRDC into a database set up by data management team at BRTU with support from DMSys. BRTU-IHRDC is responsible for data handling and record keeping.

The statistical analysis of the data and the preparation of tables and listings for final reports will be performed by the project statistician at BRTU-IHRDC with the help of oustside specialist statisticians.

10.2 Primary tests

Safety and Tolerability
- Occurrence of local and systemic adverse events
- Occurrence of clinically significant hematological and biochemical abnormalities

Immunogenicity
- ELISA for antibody titers against PEV301T and PEV302T

10.3 Secondary tests

- Influenza A ELISA/HIT
- Parasite growth/invasion inhibition assays
- IgG isotyping
- CMI: Flow cytometry for T cell phenotype, cytokine and subset analysis, quantitative real-time PCR for HLA-DRB haplotyping and T cell function
- Western Blotting and IFA for antibody titers cross-reactive with *P. falciparum* parasites (blood stages and sporozoites, respectively)

10.4 Statistical hypothesis

The combination of PEV301T + PEV302T is safe for inoculation into semi-immune subjects. No serious adverse events attributable to either vaccine occur in the study population. The vaccine formulation boosts specific immune responses in volunteers with pre-existing immunity.

10.5 Sample size calculation

The sample size of this pilot study is determined by the requirement to determine safety and immunogenicity of the combination (PEV3B). A sample size of 40 children constitute a reasonable sample size to estimate the incidence of frequent AEs with an acceptable accuracy, and to assess the boosting effect, allowing for dropouts,.

10.6 Statistical analysis

Demographic data of each study group will be tabulated.

Safety data:

Listings will be made of the safety data collected at each time point.

Descriptive statistics will be used to analyze adverse events (AEs) including intercurrent illnesses. The number of AEs and their severity will be reported using frequency tables. With frequently occurring (10 or more) event types, effects of vaccine will be tested by recording the presence/absence of the event in each patient and using logistic regression models to test for differences between vera and controls. Adults and children will be treated separately

Immunogenicity data:

Immunological data for each time point will be analyzed separately.

a) Descriptive statistics (minimum, maximum, median, geometric mean, arithmetic mean and quartiles) will be computed for each immunological measure and each time point, separately for adults and children and for vera and controls.
b) For each volunteer, the ratio of the immunological measure to that assessed at baseline (during screening) will be computed. Descriptive statistics for these ratios (minimum, maximum, median, geometric mean, and quartiles) will be computed for each immunological measure and each time point, separately for each group.
c) Wilcoxon tests will be used to compare the immunological measures between vera and controls for each immunological measure at each time point, separately for adults and children.

10.7 Final analysis

The final analysis will be performed after evaluation of the data one year after 1st injection.

10.7.1 Safety evaluation

Safety of the injected study materials will be determined as the incidence of adverse events and the occurrence of significant clinical, hematological and biochemical abnormalities during the procedure and at the intervals indicated in the schedule of assessments. Listings will be made of the safety data collected at each time point.

No serious adverse events attributable to either vaccine are expected.

10.7.2 Immunogenicity evaluation

The criteria for the evaluation of immunogenicity of the combination (PEV3B) of PEV301T + PEV302T will be the result of the following assays:

- ELISA for antibody titers against PEV301T and PEV302T, performed by Pevion Biotech Ltd.
- Western Blotting and IFA for antibody titers crossreactive with *P. falciparum* parasites (blood stages and sporozoites, respectively), performed by STI

In addition, the following evaluations will be made (but not included in the final study report):

- Parasite growth/invasion inhibition assays, performed at STI
- Isotyping, performed at STI
- Influenza ELISA/HIT, performed at Pevion Biotech Ltd.
- Flow cytometry for T cell phenotype, cytokine and subset analysis, performed at IHRDC

- Quantitative Real-Time PCR for HLA-DRB haplotyping and T cell function, performed at STI

10.8 Interim analysis

An interim analysis will be performed after obtaining the results of day 120, one month following the second injection of vaccine.

10.9 Criteria for inclusion in the analysis

Safety data analysis will be performed on all subjects who received at least one dose of study vaccine/Inflexal, and for whom at least one set of safety follow-up data is available (i.e. safety population).

Subjects will be included in the per-protocol analysis of immunogenicity if they fulfill the following criteria (i.e. per protocol analysis):

- All inclusion/exclusion criteria were respected
- They received the vaccine they were randomized to receive
- They received a second dose of the vaccine
- Allowed intervals between injections were respected
- Allowed intervals between blood sampling schedules were respected
- No forbidden vaccine or concomitant medication was taken

Subjects will be included in the intention-to-treat analysis of immunogenicity if they received at least one dose of study vaccine/Inflexal, and for whom at least one blood sample was taken.

11 Ethics and regulatory considerations

The study will be conducted according to Good Clinical Practice, the Declaration of Helsinki (Protocol Appendix A), Directive 2001/20/EC, ICH-GCP Guidelines E6, Institutional Review Boards (IRB)/ Independent Ethics Committees (IEC) and national Guidelines of Tanzania.

11.1 Independent Ethics Committee (IEC) / Institutional Review Board (IRB)

Good Clinical Practice requires that the clinical protocol, any protocol amendments, the Investigator's Brochure, the Informed Consent, and all other forms of subject information related to the study and any other necessary documents be reviewed by an Independent Ethics Committee (IEC) or Institutional Review Board (IRB).

The documents stated above will be submitted to the Ethikkommission beider Basel (EKBB), to the Institutional Review Board of the Ifakara Health Research and Development Center and to the National Institute of Medical Research Review Board for approval.

11.2 Informed consent

The Investigator or his/her representative will obtain written informed consent from each subject enrolled in the study, in accordance with the current version of the Declaration of Helsinki, the current version of the ICH guidelines and the laws and regulations of the country in which the investigation is being conducted.

The Ethics Committee/IRB must approve the informed consent document to be used by the Investigator. It is the responsibility of the Investigator to assure that the patient (or guardian or legal representative) has signed the Informed Consent before any study related

procedures are performed. In case a patient is illiterate, an impartial witness should be present during the entire consent procedure

For Information and Informed Consent Form see Appendix 2 and 3, respectively.

11.3 Benefits and Risks

Volunteers are unlikely to benefit directly from participation in this study. However, it is hoped that the information gained from this study will contribute to the development of a safe and effective malaria vaccine for the Tanzanian population. The general risks to participants in this Phase I study are associated with phlebotomy and with vaccination. The volume of blood drawn over the study should not compromise these otherwise healthy subjects. Potential risks include the following:
Local reactions: Mild tenderness, bruising, or fainting may result from venipuncture. An inflammatory reaction as manifested by redness, swelling, and/or tenderness may occur at the site of vaccine injection, although it has never been observed in the 46 volunteers in Switzerland. Absence of local adverse besides pain makes this candidate vaccine the safest one tested so far in malaria vaccine development history.
Systemic Reactions: Systemic reactions to immunization could theoretically occur, and include a flu-like illness with low-grade fever, chills and malaise. However, experience to date with other CSP vaccines suggests that if such reactions occur, they resolve in 12 days without therapy or limitation of daily activity. Serum sickness reactions due to deposition of antigen-antibody complexes, or idiosyncratic immune responses not dependent on immune complexes could theoretically develop resulting in damage to organs, such as the liver or kidney. Such immune-mediated reactions have not been reported to date after malaria vaccines. Temporary ascending paralysis, the Guillain-Barré syndrome, may occur with any vaccine, although it is very rare.
Allergic Reactions and Anaphylaxis: As with any vaccine, allergic reactions are possible. A variety of synthetic and recombinant malaria peptide vaccines have induced systemic allergic responses, e.g., urticaria and anaphylaxis. The allergic reactions occurred after 2-3 vaccinations administered at intervals of 1 month or more, vaccines were formulated with Alhydrogel or QS-21 adjuvants, and high antigen concentrations (200 µg - 2000 µg) were inoculated with each dose. Reactions were occasionally associated with development of antigen-specific IgE antibody. Again, the long history of other virosome-formulated vaccines presently commercialized makes the present vaccine safer than other candidates currently in development, at least for the adjuvant/immunomodulator component.
Adventitious Agents: Each lot of vaccine is tested for sterility and for either endotoxin content or pyrogenicity. The formulated product is released for use in humans when the release criteria are met, which suggests that the product is free of known microbial contamination that could infect humans. Nevertheless, the risk of unknown microbe is always present.

12 Administrative Matters

To comply with Good Clinical Practice important administrative obligations relating to investigator responsibilities, monitoring, archiving data, audits, confidentiality and publications must be fulfilled.

12.1 Protocol and Investigators Brochure (IB)

As it is essential that the study is carried out exactly in accordance with the terms of the protocol, the clinicians will familiarize themselves fully with it to be able to conduct the study in the manner specified. Any amendments, revisions or modifications to the protocol must be in writing and agreed upon by all parties, including ethics committees prior to their implementation.

Pevion Biotech Ltd. will supply the investigators with an Investigator's Brochure (IB) which describes the vaccines being tested and its known adverse effects.

12.2 Confidentiality

Either prior to or during the course of the study, Pevion Biotech Ltd. / IHRDC, PMU/STI or their representatives will provide the investigators and persons delegated by them with confidential information, for example, but not limited to, the protocol and the IB. The information may not be disclosed to anyone else without prior approval from Pevion Biotech Ltd. / BRTU-IHRDC, PMU/STI in writing. This obligation of confidentiality shall survive the completion or early termination of the study.

12.3 Documentation and Material Supplies

All supplies provided to the investigators for the purpose of carrying out the study are supplied only for the purpose of the study and must not be used for any other purpose whatsoever. The investigators or (a) person(s) delegated by him is/are responsible for the security and accountability of all supplies. All such supplies, if not used during the course of the study and not forming a part of the documentation required to be retained by the investigators, must be returned to Pevion Biotech Ltd. / PMU/STI at the conclusion of the study.

12.4 Financial compensation to the participants

There willl be no financial compensation. All volunteers will receive a small amount of money to cover transport expenses and loss of other financial benefits.

12.5 Monitoring

The study will be coordinated and monitored by the Pharmaceutical Medicine Unit (PMU) of the Swiss Tropical Institute (STI). Its representatives will be allowed access to all information resulting from this study and Pevion Biotech Ltd. will have an unrestricted right to use such information. The study monitor will have access to all study-related source documentation. The subjects' confidentiality will be respected as required by local law.

12.6 Source Documents and Case Report Forms (CRFs)

A CRF must be completed for each subject who enters the study and it is the investigators responsibility to ensure that the CRFs are complete and accurate. Each case record form must be completed using black or blue ball point pens. Any mistakes must be crossed through with a single line, not obliterated or corrected with liquid paper (e.g. Tipp-Ex®), the correction added and signed and dated by the the investigators.
Independent case histories for each subject and source documents must be maintained to support the data entered on the CRF and the investigators commit to make all subject medical records and other source documents, which are pertinent to the study available to Pevion Biotech Ltd. / PMU/STI and/or health authority representatives upon request. All subject records must be kept in a secure place for fifteen (15) years.

12.7 Quality assurance audit

Independent auditors designated by Pevion Biothech Ltd. may conduct a systematic examination of study related activities, documents and selected sites to assess whether the evaluated study activities were conducted, and data were recorded, analyzed and accurately reported according to approved protocol, standard operating procedures current Good Clinical Practice, and the applicable regulatory requirements. Audit observations and findings

will be documented and communicated to appropriate study personnel and management. A corrective and preventative action plan will be requested and documented in response to any audit observations. Audit reports and responses to audit observations must be returned to the responsible auditor. When required by regulations, the auditor will provide an audit certificate.

12.8 Compensation for medicine-induced injury

Pevion Biotech Ltd. assumes liability for and will indemnify all injuries that occur to trial subjects or subjects whenever a causal relationship can be established between the event and the clinical trial procedure or the trial substance under study if the following can be demonstrated:

a. The event resulted from a trial substance, provided that the substance was administered according to the approved trial protocol PMAL03.

b. The event arose in association with the use of comparative substances used legitimately as part of the trial protocol.

c. The event occurred as a consequence of non-routine / study specific diagnostic procedures performed according to the trial protocol.

d. The event resulted from therapeutic or diagnostic measures legitimately required as a consequence of unexpected events caused by the trial substance, by comparative medication, or by diagnostic procedures called for by the trial protocol.

Pevion Biotech Ltd is not liable for events that occur solely as a consequence of the underlying illness of the trial subject or subject, or for events resulting from diagnostic or therapeutic measures not specifically required by the trial protocol, or for events resulting from negligence (including failure to act according to accepted medical practice, or to comply strictly with the protocol or the terms of this Agreement) of the investigators or any other involved and/or related clinical staff and facilities.

This indemnity provided by Pevion Biotech Ltd shall further apply as follows:

a. Pevion Biotech Ltd. is to be informed as soon as possible of any complaint, action or suit of proceeding giving rise to the right of indemnification, and the investigators agree to co-operate fully with Pevion Biotech Ltd in the defense or disposition of all such cases.

b. Pevion Biotech Ltd will be permitted, at its costs and discretion, to handle and control the defense or disposition of all such cases.

c. No case will be settled without the prior written consent of Pevion Biotech Ltd.

12.9 Early termination

It is the intention of all that this study is carried out to its conclusion, but the investigators must be aware that for a number of reasons, the study may need to be stopped prior to their conclusion. Pevion Biotech Ltd. or STI, therefore, reserves the right to terminate this Agreement:

a. immediately upon a substantial breach of the terms either of the Agreement or of the conduct of the protocol;

b. in the event of irregularities in the method by which the study is carried out and although capable of being rectified, are not rectified within thirty (30) days of notice from Pevion Biotech Ltd. / PMU/STI requiring this.

c. immediately, if this is necessary in the interests of health and safety of the study subjects, or as a result of an order of any government authority or court of law.

In the event of early termination, the investigators will cease use of the investigational drug immediately. All CRFs outstanding must be completed and returned to Pevion Biotech Ltd. together with completed drug inventory, records and remaining trial material.

12.10 Publication of data and protection of trade secrets

The investigators agree to submit all manuscripts or abstracts to Pevion Biotech Ltd. / STI prior to submission. This allows Pevion Biotech Ltd. to protect proprietary information and to provide comments based on information from other studies that may not yet be available to the authors of the manuscript. The investigators will collaborate with the study statistician for preparation of trial data analyses intended to be used in the publication(s) of the study.

The following points need to be considered:

a. Without principal investigators' written consent, Pevion Biotech Ltd may not make reference, either directly or indirectly, in a commercial publication, to the investigators' name or institution, or any of its employees in which the investigators performed the present trials, connected with the research and its results.

b. Pevion Biotech Ltd. may not use the investigators' name or the name of the IHRDC or its employees connected to the research or to the institution in which the investigators performed the present trial in its commercial publications as recommences of quality and/or of the finished product and/or of the drug and the efficacy of its use.

Nothing in the aforementioned limitations in clauses a.-b. will prevent Pevion Biotech Ltd. from quoting from articles, provided that the scientific source of data (scientific conventions, scientific newspapers) is mentioned.

12.11 Intellectual property rights

Data resulting from this study shall be the sole property of Pevion Biotech Ltd. and STI. Should any inventions/improvements result from this study, Pevion Biotech Ltd. shall be entitled to file in its own name relevant patent applications, and the said inventions and improvements will become and remain the sole property of Pevion Biotech Ltd. The investigators agree to provide Pevion Biotech Ltd. with all requested assistance necessary for obtaining any patents, including execution of legal documents. It is understood that any publication is withheld until patent application is filed.

12.12 Entry violations

All subjects, including those who enter the study but are found to violate the entry criteria <u>must be followed</u> to the end of the study.

13 References

1. Bisang, C, Jiang L, Freund, E, Emery, F, Bauch, C, Matile, H, Pluschke, G, Robinson, J. A. Synthesis, conformational properties, and immunogenicity of a cyclic template-bound peptide mimetic containing an NPNA motif from the circumsporozoite protein of *Plasmodium falciparum.* J Am Chem Soc 1998;120:7439 – 7449

2. Pöltl-Frank, F, Zurbriggen, R, Helg, A, Stuart, F, Robinson, J, Glück, R, Pluschke, G Use of reconstituted influenza virus virosomes as an immunopotentiating delivery system for a peptide-based vaccine. Clin Exp Immunol 1999;117:496-503

3. Pfeiffer, B, Moreno, R, Moehle, K, Zurbriggen, R, Glück, R, Pluschke, G, and Robinson, J A, Application of protein epitope mimetics in vaccine design. Chimia 2001;55:334 - 339

4. Moreno, R, Jiang, L, Moehle, K, Zurbriggen, R, Glück, R, Robinson, JA and Pluschke, G Exploiting conformationally constrained peptidomimetics and an efficient human-compatible delivery system in synthetic vaccine design. Chem Bio Chem 2001;2:838 - 843

5. Pfeiffer B, Peduzzi E, Moehle K, Zurbriggen R, Glück R, Pluschke G and Robinson JA A virosome-mimotope (Viro-Tope) approach to synthetic vaccine design and optimization: synthesis, conformation and immune recognition of a new potential malaria vaccine candidate. Angewandte Chemie 2003;42:2368 – 2371

6. Mueller MS, Renard A, Boato F, Vogel D, Naegeli M, Zurbriggen R, Robinson JA, and Pluschke G Induction of Parasite Growth Inhibitory Antibodies by a Virosomal Formulation of a Peptidomimetic of Loop-I from Domain III of *Plasmodium falciparum.* Infect Immun 2003;71:4758-4779.

7. Genton B, Pluschke G, Degen L, Kammer AR, Westerfeld N, Okitsu SL, Schroller S, Vounatsou P, Mueller MM, Tanner M, Zurbriggen R. A randomized placebo-controlled phase Ia malaria vaccine trial of two virosome-formulated synthetic peptides in healthy adult volunteers. Plos One 2007, in press.

8. Okitsu SL, Silvie O, Westerfeld N, Curcic M, Mueller MS, Sauerwein R, Genton B, Mazier D, Kammer AR, Zurbriggen R, Pluschke G. A virosomally formulated malaria peptide candidate vaccine elicits a long-lasting Plasmodium falciparum sprozoite-inhibitory antibody response in human volunteers. Submitted to Plos Clinical Trials.

9. Masurel N. Influenza A and B antibody status in Tanzania. Trans. R. Soc. Trop. Med. Hyg. 1987;81:75-9.

Appendix 1: Helsinki Declaration

WORLD MEDICAL ASSOCIATION DECLARATION OF HELSINKI

Ethical Principles for Medical Research Involving Human Subjects
Adopted by the 18th WMA General Assembly, Helsinki, Finland, June 1964, and amended by the
29th WMA General Assembly, Tokyo, Japan, October 1975
35th WMA General Assembly, Venice, Italy, October 1983
41st WMA General Assembly, Hong Kong, September 1989
48th WMA General Assembly, Somerset West, Republic of South Africa, October 1996
and the 52nd WMA General Assembly, Edinburgh, Scotland, October 2000
Note of Clarification on Paragraph 29 added by the WMA General Assembly, Washington 2002
Note of Clarification on Paragraph 30 added by the WMA General Assembly, Tokyo 2004

A. INTRODUCTION

1. The World Medical Association has developed the Declaration of Helsinki as a statement of ethical principles to provide guidance to physicians and other participants in medical research involving human subjects. Medical research involving human subjects includes research on identifiable human material or identifiable data.
2. It is the duty of the physician to promote and safeguard the health of the people. The physician's knowledge and conscience are dedicated to the fulfillment of this duty.
3. The Declaration of Geneva of the World Medical Association binds the physician with the words, "The health of my patient will be my first consideration," and the International Code of Medical Ethics declares that, "A physician shall act only in the patient's interest when providing medical care which might have the effect of weakening the physical and mental condition of the patient."
4. Medical progress is based on research which ultimately must rest in part on experimentation involving human subjects.
5. In medical research on human subjects, considerations related to the well-being of the human subject should take precedence over the interests of science and society.
6. The primary purpose of medical research involving human subjects is to improve prophylactic, diagnostic and therapeutic procedures and the understanding of the aetiology and pathogenesis of disease. Even the best proven prophylactic, diagnostic, and therapeutic methods must continuously be challenged through research for their effectiveness, efficiency, accessibility and quality.
7. In current medical practice and in medical research, most prophylactic, diagnostic and therapeutic procedures involve risks and burdens.
8. Medical research is subject to ethical standards that promote respect for all human beings and protect their health and rights. Some research populations are vulnerable and need special protection. The particular needs of the economically and medically disadvantaged must be recognized. Special attention is also required for those who cannot give or refuse consent for themselves, for those who may be subject to giving consent under duress, for those who will not benefit personally from the research and for those for whom the research is combined with care.
9. Research Investigators should be aware of the ethical, legal and regulatory requirements for research on human subjects in their own countries as well as applicable international requirements. No national ethical, legal or regulatory

requirement should be allowed to reduce or eliminate any of the protections for human subjects set forth in this Declaration.

B. BASIC PRINCIPLES FOR ALL MEDICAL RESEARCH

10. It is the duty of the physician in medical research to protect the life, health, privacy, and dignity of the human subject.
11. Medical research involving human subjects must conform to generally accepted scientific principles, be based on a thorough knowledge of the scientific literature, other relevant sources of information, and on adequate laboratory and, where appropriate, animal experimentation.
12. Appropriate caution must be exercised in the conduct of research which may affect the environment, and the welfare of animals used for research must be respected.
13. The design and performance of each experimental procedure involving human subjects should be clearly formulated in an experimental protocol. This protocol should be submitted for consideration, comment, guidance, and where appropriate, approval to a specially appointed ethical review committee, which must be independent of the investigator, the sponsor or any other kind of undue influence. This independent committee should be in conformity with the laws and regulations of the country in which the research experiment is performed. The committee has the right to monitor ongoing trials. The researcher has the obligation to provide monitoring information to the committee, especially any serious adverse events. The researcher should also submit to the committee, for review, information regarding funding, sponsors, institutional affiliations, other potential conflicts of interest and incentives for subjects.
14. The research protocol should always contain a statement of the ethical considerations involved and should indicate that there is compliance with the principles enunciated in this Declaration.
15. Medical research involving human subjects should be conducted only by scientifically qualified persons and under the supervision of a clinically competent medical person. The responsibility for the human subject must always rest with a medically qualified person and never rest on the subject of the research, even though the subject has given consent.
16. Every medical research project involving human subjects should be preceded by careful assessment of predictable risks and burdens in comparison with foreseeable benefits to the subject or to others. This does not preclude the participation of healthy volunteers in medical research. The design of all studies should be publicly available.
17. Physicians should abstain from engaging in research projects involving human subjects unless they are confident that the risks involved have been adequately assessed and can be satisfactorily managed. Physicians should cease any investigation if the risks are found to outweigh the potential benefits or if there is conclusive proof of positive and beneficial results.
18. Medical research involving human subjects should only be conducted if the importance of the objective outweighs the inherent risks and burdens to the subject. This is especially important when the human subjects are healthy volunteers.
19. Medical research is only justified if there is a reasonable likelihood that the populations in which the research is carried out stand to benefit from the results of the research.
20. The subjects must be volunteers and informed participants in the research project.
21. The right of research subjects to safeguard their integrity must always be respected. Every precaution should be taken to respect the privacy of the subject, the confidentiality of the patient's information and to minimize the impact of the study on the subject's physical and mental integrity and on the personality of the

subject.
22. In any research on human beings, each potential subject must be adequately informed of the aims, methods, sources of funding, any possible conflicts of interest, institutional affiliations of the researcher, the anticipated benefits and potential risks of the study and the discomfort it may entail. The subject should be informed of the right to abstain from participation in the study or to withdraw consent to participate at any time without reprisal. After ensuring that the subject has understood the information, the physician should then obtain the subject's freely-given informed consent, preferably in writing. If the consent cannot be obtained in writing, the non-written consent must be formally documented and witnessed.
23. When obtaining informed consent for the research project the physician should be particularly cautious if the subject is in a dependent relationship with the physician or may consent under duress. In that case the informed consent should be obtained by a well-informed physician who is not engaged in the investigation and who is completely independent of this relationship.
24. For a research subject who is legally incompetent, physically or mentally incapable of giving consent or is a legally incompetent minor, the investigator must obtain informed consent from the legally authorized representative in accordance with applicable law. These groups should not be included in research unless the research is necessary to promote the health of the population represented and this research cannot instead be performed on legally competent persons.
25. When a subject deemed legally incompetent, such as a minor child, is able to give assent to decisions about participation in research, the investigator must obtain that assent in addition to the consent of the legally authorized representative.
26. Research on individuals from whom it is not possible to obtain consent, including proxy or advance consent, should be done only if the physical/mental condition that prevents obtaining informed consent is a necessary characteristic of the research population. The specific reasons for involving research subjects with a condition that renders them unable to give informed consent should be stated in the experimental protocol for consideration and approval of the review committee. The protocol should state that consent to remain in the research should be obtained as soon as possible from the individual or a legally authorized surrogate.
27. Both authors and publishers have ethical obligations. In publication of the results of research, the investigators are obliged to preserve the accuracy of the results. Negative as well as positive results should be published or otherwise publicly available. Sources of funding, institutional affiliations and any possible conflicts of interest should be declared in the publication. Reports of experimentation not in accordance with the principles laid down in this Declaration should not be accepted for publication.

C. ADDITIONAL PRINCIPLES FOR MEDICAL RESEARCH COMBINED WITH MEDICAL CARE

28. The physician may combine medical research with medical care, only to the extent that the research is justified by its potential prophylactic, diagnostic or therapeutic value. When medical research is combined with medical care, additional standards apply to protect the patients who are research subjects.
29. The benefits, risks, burdens and effectiveness of a new method should be tested against those of the best current prophylactic, diagnostic, and therapeutic methods. This does not exclude the use of placebo, or no treatment, in studies where no proven prophylactic, diagnostic or therapeutic method exists.1
30. At the conclusion of the study, every patient entered into the study should be assured of access to the best proven prophylactic, diagnostic and therapeutic

methods identified by the study.2
31. The physician should fully inform the patient which aspects of the care are related to the research. The refusal of a patient to participate in a study must never interfere with the patient-physician relationship.
32. In the treatment of a patient, where proven prophylactic, diagnostic and therapeutic methods do not exist or have been ineffective, the physician, with informed consent from the patient, must be free to use unproven or new prophylactic, diagnostic and therapeutic measures, if in the physician's judgement it offers hope of saving life, re-establishing health or alleviating suffering. Where possible, these measures should be made the object of research, designed to evaluate their safety and efficacy. In all cases, new information should be recorded and, where appropriate, published. The other relevant guidelines of this Declaration should be followed.

1 Note of clarification on paragraph 29 of the WMA Declaration of Helsinki
The WMA hereby reaffirms its position that extreme care must be taken in making use of a placebo-controlled trial and that in general this methodology should only be used in the absence
of existing proven therapy. However, a placebo-controlled trial may be ethically acceptable, even if proven therapy is available, under the following circumstances:
- Where for compelling and scientifically sound methodological reasons its use is necessary to
determine the efficacy or safety of a prophylactic, diagnostic or therapeutic method; or
- Where a prophylactic, diagnostic or therapeutic method is being investigated for a minor condition and the patients who receive placebo will not be subject to any additional risk of serious or irreversible harm.

All other provisions of the Declaration of Helsinki must be adhered to, especially the need for appropriate ethical and scientific review.

2 Note of clarification on paragraph 30 of the WMA Declaration of Helsinki
The WMA hereby reaffirms its position that it is necessary during the study planning process to
identify post-trial access by study participants to prophylactic, diagnostic and therapeutic procedures identified as beneficial in the study or access to other appropriate care. Post-trial access arrangements or other care must be described in the study protocol so the ethical review
committee may consider such arrangements during its review.

9.10.2004
